# Evaluating Sex Differences in Efficacy, Safety and Pharmacokinetics in Patients Treated with Cannabis by a Metered-Dose Inhaler

**DOI:** 10.3390/ph16101426

**Published:** 2023-10-08

**Authors:** Joshua Aviram, Marek Glezerman, Eytan Hayam, Rostislav Belobrov, Shiri Procaccia, David Meiri, Elon Eisenberg

**Affiliations:** 1 Syqe Medical Ltd., Tel Aviv 6816914, Israel; 2 Faculty of Medicine and Head, Gender- and Sex Conscious Medicine, Tel Aviv University, Tel Aviv 69978, Israel; 3 Faculty of Biology, Technion-Israel Institute of Technology, Haifa 3200003, Israel; 4 Rappaport Faculty of Medicine-Technion, Israel Institute of Technology, Haifa 3525433, Israel

**Keywords:** sex, medical cannabis, medicinal cannabis, chronic pain, phytocannabinoids, adverse effects, Syqe inhaler, pharmacokinetics

## Abstract

Background: Clinical studies on medical cannabis (MC) treatment have shown sex-related differences, including higher susceptibility to adverse events among women and greater analgesia among men. Here, we used the Syqe metered-dose inhaler (MDI) and a single chemovar to analyze sex differences. Methods: A total of 1249 Israeli chronic pain patients were assessed for pain intensity, sleep and adverse events (AEs) over 240 days. Results: Following the first two weeks, no significant sex differences were found in the effectiveness or safety of MC treatment (*p* > 0.05). Inhaled Δ^9^-THC doses did not vary significantly between sexes (*p* > 0.05) except in the first month of treatment. Pain reduction and sleep improvement were similar for both sexes (*p* > 0.05). The overall rate of AEs was equal and relatively low at 10% (n = 65, 10% of women and n = 60, 10% of men; χ^2^ (1) = 0.05, *p* = 0.820). A secondary analysis of pharmacokinetic data showed no significant differences between sexes in Δ^9^-THC and its metabolite pharmacokinetics, cardiovascular measures, or AE severity (*p* > 0.05). Conclusions: Uniform MC treatment via the Syqe MDI showed no sex differences in short-term effectiveness, safety and pharmacokinetics, nor in long-term effects, under “real-life” conditions. These findings provide insights into MC treatment which may inform clinical practice and policy-making in the field.

## 1. Introduction

Chronic pain diagnoses are more common in women than in men [1]. For example, migraine and fibromyalgia are three and nine times more common in women than men, respectively [2]. Medical cannabis (MC) use is becoming more prevalent in many countries [3], most prominently for chronic non-cancer pain (CNCP) treatment [4]. Although clear evidence regarding its efficacy and safety for chronic pain are inconsistent, preclinical and clinical studies have raised the possibility for differences in the effectiveness, safety and pharmacokinetics (PK) between sexes [5,6].

The cannabis plant contains phytocannabinoids such as ∆^9^-tetrahydrocannabinol (Δ^9^-THC) and cannabidiol (CBD) that exert pharmacological effects via the endocannabinoid system, a neuromodulatory signaling system that has widespread functions [7]. Animal studies have shown that gonadal sex hormones affect the concentrations of endocannabinoid ligands in several brain areas [8,9,10]. An in vivo study in rats showed higher levels of 11-hydroxy-Δ^9^-THC in female rats, who also demonstrated antinociception to a painful stimulus that was not observed in male rats in a tail withdrawal assay [5].

A study of pain in humans [11] reported contradictory findings of superior MC analgesia in men compared to women. Our group has previously shown that women exhibited higher susceptibility to MC-related adverse events (AEs) relative to men [6]. This difference was attributed to two possible factors: a. inherited/biological sex-related differences in the response to MC and b. different MC cultivar combinations consumed by women, which consisted of different compositions of phytocannabinoids and terpenoids than those consumed by men. One additional study on the recreational use of cannabis found that women were more susceptible to Δ^9^-THC’s psychoactive effects at a lower dose than men [12]. In terms of general adverse drug reactions, it was previously described that female patients have a 1.5- to 1.7-fold increased risk of reporting AEs following drug consumption relative to male patients due to sex-related differences [13], including, but not limited to, factors such as body weight and social factors.

MC can be consumed via different routes, most commonly by inhalation (i.e., smoking/vaporization) [14], which allows a rapid onset of systemic effects. However, to the best of our knowledge, all hitherto published routes are characterized by inconsistent PK [15], analgesic and safety profiles [14]. The Syqe metered-dose inhaler (MDI) version 1.1 (Trade name SyqeAir, Syqe Medical, Tel Aviv-Yafo, Israel) is a selective-dose MC inhaler that provides a consistent and precise Δ^9^-THC concentration in the blood following inhalation of low doses of MC [16]. Previously published PK studies of MC administered using an earlier, bioequivalent version of the MDI showed a narrow variability in C_max_ between patients and a dose–response analgesic effect [16,17]. We recently published a cohort study on preliminary data from the data set described in the current study in which we analyzed prospective “real-life” data of 143 patients using the MDI with an average monthly dose of 1.21 g. The patients achieved a similar effectiveness to that of high-dose MC (i.e., 20–50 g/month) [18], but with a superior safety profile [19].

Here, in order to assess sex differences in effectiveness, safety and PK, while eliminating potential variability between women and men in MC cultivar combinations, we performed a secondary analysis of data from two published studies with the Syqe MDI [16,17] with a focus on the differences between men and women. First, we analyzed updated data collected from the “Real-life” cohort described above [19]. Then, our data from two previously published PK studies [16,17] were combined and reanalyzed.

## 2. Results

### 2.1. Real-World Evidence Data

#### 2.1.1. Sample

**Figure 1:**
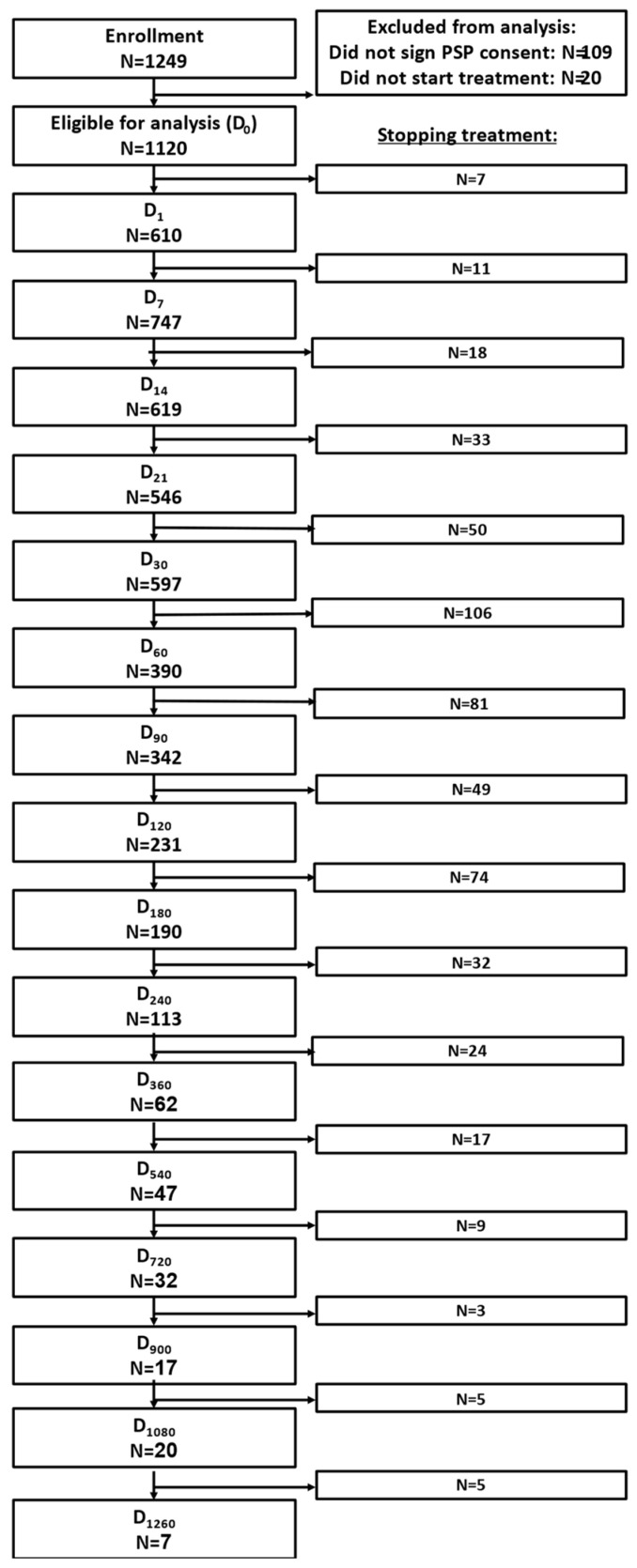
CONSORT 2010 Flow Diagram (number of patients). D, day from the first use of the inhaler. Only patients who reported their NPS score or daily dose were included in each visit’s analysis.

A total of 1249 patients from Israel who were treated with MC via the MDI were analyzed in this study. Of them, 109 patients did not sign the patient support program (PSP) consent form and 20 additional patients chose not to initiate treatment following enrollment. Although safety analyses were performed for all patients (Figure 1), efficacy analyses were only conducted on the 1120 patients that signed the consent form and initiated the treatment.

All 1120 patients who signed the consent form were included in the current analysis. Of them, 524 (47%) patients stopped treatment at different times for various reasons during the 1260 days of treatment, and 409 (37%) stopped at six months (Table S1). Notably, of patients receiving full reimbursement for the treatment (n = 185), the attrition rate at six months was 22%. The total attrition rate during the 1260 days was not significantly different between men and women. Analysis of the specific reasons revealed that men’s attrition due to switching to another administration route was more prevalent (n = 34, 12%) than women’s attrition (n = 51, 8%) (χ^2^_(1)_ = 44.16, *p* = 0.04) when all time points were combined (Table S1). However, there were no differences in the overall attrition reasons at each follow-up time point (Table S2) or in the specific reasons at each follow-up time point (Table S3) (*p* > 0.05).

##### Patient Characteristics

At baseline (D_0_) (Table 1), of the 1120 patients that were enrolled and analyzed, 607 (54%) were women. Age and BMI were not statistically different between the sexes (*p* > 0.05). None of the women were pregnant or breastfeeding at enrollment nor throughout the study. About half of the patients had previous experience with MC treatment prior to using the inhaler (n = 604, 54%). Previous use of MC inflorescence by men was higher (n = 144, 28%) than by women (n = 116, 19%) (χ^2^_(1)_ = 12.52, *p* < 0.001) and men had consumed significantly larger doses of MC (23.0 ± 13.1 gr/M) compared to women (19.6 ± 10.8 gr/M) (χ^2^_(1)_ = 9.9, *p* < 0.005).

Most patients (n = 919, 82%) were prescribed MC treatment due to chronic pain. An additional 98 patients (9%) had chronic pain as the non-primary MC indication diagnosis, bringing the total to 91% (n = 1017) of the patients with a chronic pain diagnosis. Chronic pain indication, and specifically, chronic neuropathic pain, were more frequent among men (n = 353, 69%) than among women (n = 345, 57%) (χ^2^_(1)_ = 4.41, *p* = 0.04 and χ^2^_(1)_ = 16.47, *p* ≤ 0.001, respectively). Other, more comprehensive MC indication/comorbidity comparisons between the sexes are presented in Table S4. Notably, although the etiologies and diagnoses were very heterogenous, some differences between the sexes were apparent, probably due to exclusive or predominant sex-related diagnoses, such as endometriosis, osteoarthritis, rheumatoid arthritis, migraine and fibromyalgia in women, and post-traumatic stress disorder (PTSD) nerve injury and phantom pain in men. 

Comorbidities were reported by 793 patients (71%), with similar rates (*p* > 0.05) for women (n = 435, 72%) and men (n = 358, 70%) (Table S5). Men had significantly higher rates of diabetes, hyperlipidemia, cardiac disease and ischemic heart disease than women, while women had significantly higher rates of hypothyroidism, osteoporosis, asthma and depression (*p* < 0.05).

#### 2.1.2. Inhaler Treatment Characteristics

**Figure 2:**
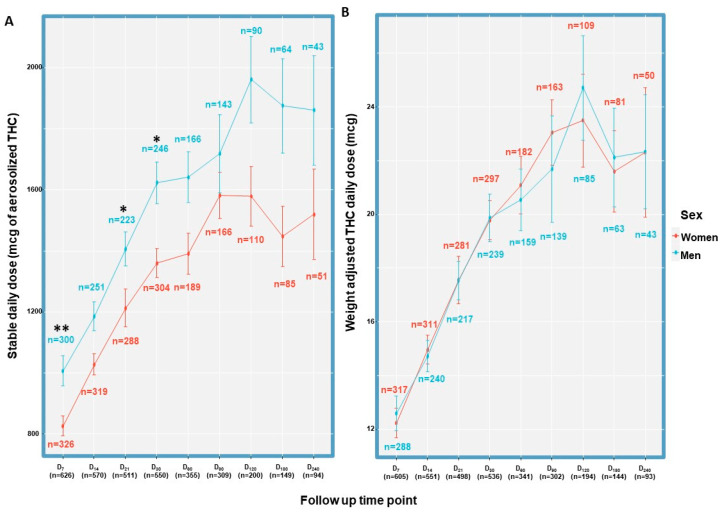
Sex comparison of Δ^9^-THC doses during MDI treatment. Unadjusted (**A**) and weight-adjusted (**B**) Δ^9^-THC doses in women and men. D, day; *, *p* < 0.05 and **, *p* < 0.01 in a two-sample Kolmogorov–Smirnov test between the sexes in the specific time point; Δ^9^-THC, ∆-9-tetrahydrocannabinol. Sample sizes are different between A and B due to missing data on weight; error bars represent standard deviation.

The average daily aerosolized Δ^9^-THC dose started at D_1_ with a mean ± SD of 579 ± 374 mcg, increasing linearly to 1500 ± 947 mcg at D_30_, then plateaued by D_90_ to a daily dose of 1600 ± 1300 mcg, which remained stable and similar in all follow-up time points. When examining the raw daily Δ^9^-THC doses in mcg, at a few initial follow-up time points (D_7_, D_21_ and D_30_), men consumed significantly higher daily doses relative to women (χ^2^_(1)_ = 0.15, *p* < 0.01, χ^2^_(1)_ = 0.13, *p* < 0.05 and χ^2^_(1)_ = 0.12, *p* < 0.05, respectively) (Figure 2A). However, weight-adjusted daily doses of Δ^9^-THC did not differ between men and women at any of the follow-up time points (*p* > 0.05) (Figure 2B). Following the achievement of a stable dose, only a minority of the patients (7–11% at all time points) required rescue inhalations for breakthrough pain. 

#### 2.1.3. Treatment Effectiveness

**Figure 3:**
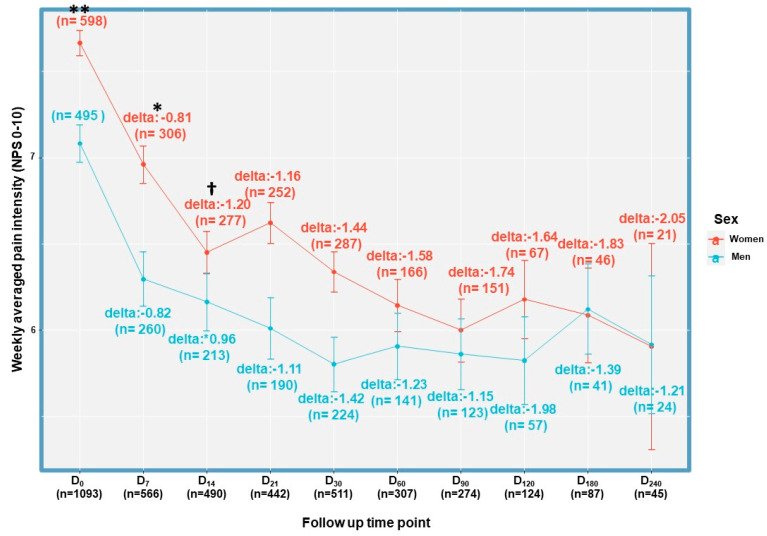
Sex comparison of pain reduction during MDI treatment. D, day; NPS, numerical pain scale; BL, baseline; *, *p* < 0.05, **, *p* < 0.01 in a two-sample Kolmogorov-Smirnov test between the sexes at the specific time point; †, *p* < 0.05 in a two-sample Kolmogorov-Smirnov test between the sexes for the difference from BL at the specific time point. Error bars represent standard deviation.

At D_0_ and in the first follow-up time point (D_7_), the average weekly pain intensity was statistically higher among women than men (χ^2^_(1)_ = 0.11, *p* < 0.01 and χ^2^_(1)_ = 0.14, *p* < 0.01, respectively). At the following time points, pain intensity was similar between the sexes (*p* > 0.05). The mean change in pain intensity from D0 was similar at most follow-up time points (*p* > 0.05), except for D_14_, when women exhibited superior reduction in pain intensity (−1.20 ± 1.60 NPS points) compared to men (−0.96 ± 1.90 NPS points) (χ^2^_(1)_ = 0.13, *p* < 0.05) (Figure 3). The pain reduction response comparison data are presented in Results S1.

##### Evaluation of Sleep Latency, Duration and Quality

**Figure 4:**
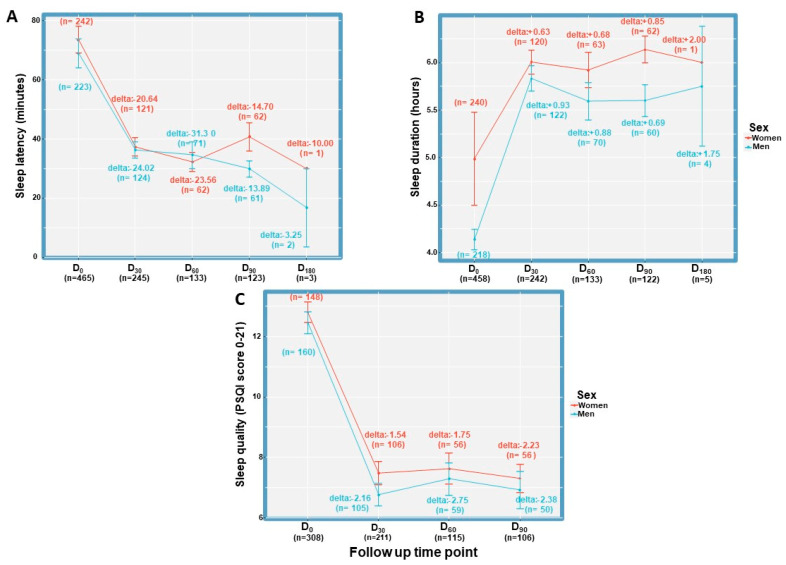
Sex comparison of sleep parameters during MDI treatment. (**A**) Sex comparison of sleep latency during MDI treatment, (**B**) Sex comparison of sleep duration during MDI treatment, (**C**) Sex comparison of sleep quality during MDI treatment. D, day; PSQI, Pittsburgh Sleep Quality Index. Error bars represent standard deviation.

No sex-related differences were found in sleep parameters at all time points (*p* > 0.05). Overall, sleep latency improved (i.e., shortened), sleep duration improved (i.e., lengthened and sleep quality improved (Figure 4).

#### 2.1.4. Treatment Safety

There were no differences in the distribution of AE reports in general between men and women (~10% for both sexes) (*p* > 0.05). For both sexes, AEs were reported significantly more frequently at the initiation of the treatment and gradually decreased. There were no differences in the distribution of AE reports between males and females at the specific follow-up time points.

Among the 1249 patients that were enrolled in MDI treatment from September 2019 to January 2023, 415 adverse events reports were filed (some occurring in the same patients). Of these, 29 were reports of the passing away of the patient unrelated to MDI treatment and an additional 159 reports were deemed unrelated to the device or to the MC administered via the inhaler by Syqe’s nursing director. Thus, 227 treatment-related AE reports were analyzed. These were reported by 125 (10%) of the 1249 enrolled patients, with at least one AE in at least one individual follow-up time point (n = 65, 10% of women and n = 60, 10% of men; χ^2^_(1)_ = 0.05, *p* = 0.820) (Table 2). Of note, none of these AEs was designated as a severe AE.

There were no differences in the rates of AEs between the sexes in any of the body systems/SOCs (*p* > 0.05). The most frequent AE PTs were dizziness (excluding vertigo) (n = 45, 4%), cough (n = 32, 3%), headache (n = 31, 2%), sleepiness (n = 27, 2%) and sore throat (n = 25, 2%). The frequency of all other AEs was <1%. Specific AEs were not significantly different between the sexes (see Table S6 by MedDRA PT).

Most AEs were short-term, and all resolved spontaneously without intervention. None of the reported AEs were caused by malfunction of the MDI.

### 2.2. PK Studies

#### 2.2.1. Description

To support the presented real-world data that were gathered in an open-label design, we performed a secondary analysis for differences between the sexes of our two previously published clinical studies. These were performed under double-blinded conditions and included assessments of blood PK and objective measurements such as pulse and blood pressure.

#### 2.2.2. Sample

We re-analyzed patients that were administered a single uniform dose of 1000 mcg Δ^9^-THC: 8 patients from the study by Eisenberg et al. (2014) [17] and 25 patients from the study by Almog et al. (2020) [16]. Notably, patients in this secondary analysis were overall younger than the patients in the real-world data sample presented above, but with similar BMIs. In the combined pharmacokinetic data, 18 (72%) had comorbidities, with a higher rate for women (n = 5, 83%) than for men (n = 13, 68%) (Table S7). All patients had MC experience via smoking and vaporization as it was part of the inclusion criteria. 

#### 2.2.3. PK Characteristics

**Figure 5:**
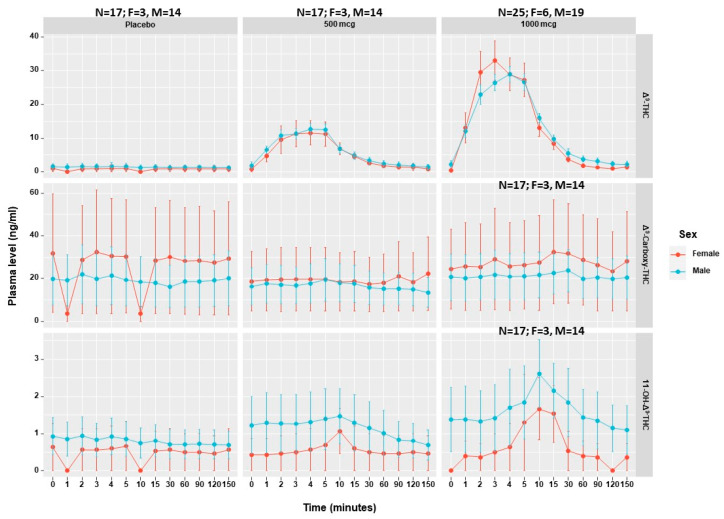
Sex differences in Δ^9^-THC and its metabolites’ pharmacokinetics. Δ^9^-THC, tetrahydrocannabinol; 11-nor-9-carboxy-Δ^9^-tetrahydrocannabinol; 11-OH-Δ^9^-THC, 11-nor-9-carboxy-Δ^9^-tetrahydrocannabinol; M, men; W, women. Error bars represent standard deviation.

In the combined pharmacokinetic data, no sex-related differences were found in the blood levels of Δ^9^-THC and its metabolites, 11-hydroxy-Δ^9^-THC and Δ^9^-Carboxy-THC, at any of the time points (*p* > 0.05) (Figure 5). Nonetheless, we found that a higher dose of 1000 mcg Δ^9^-THC produced almost double the plasma levels of Δ^9^-THC following a 500 mcg Δ^9^-THC dose at C_max_. This was not the same for Δ^9^-THC metabolites (Δ^9^-Carboxy-THC and 11-OH-Δ^9^-THC) which were found to be relatively stable, regardless of the administered dose of Δ^9^-THC (Figure S1). Notably, Δ^9^-THC metabolites were also detected and displayed for the placebo arm as all patients were assessed following at least 12 h after their regular MC treatment. The individual patients’ pharmacokinetic responses, differentiated by sex, are displayed in Figure S2.

#### 2.2.4. Cardiovascular Measurements

There were no significant differences between males and females in blood pressure and heart rate (Figures S3 and S4).

#### 2.2.5. Treatment Safety

**Figure 6:**
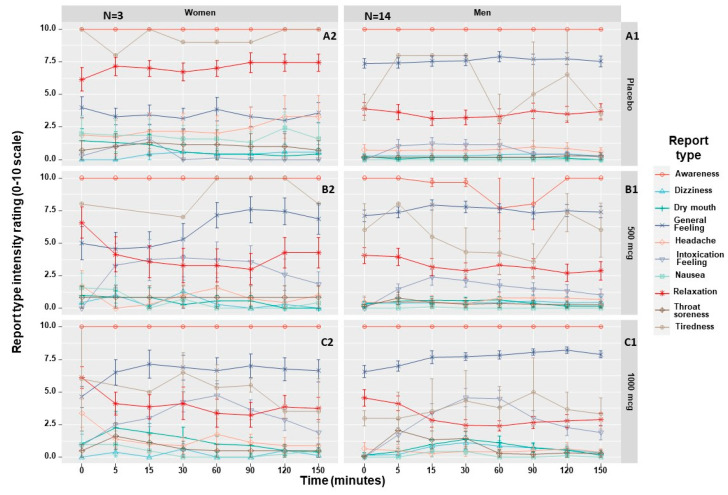
Sex comparison of adverse events and positively perceived effects following a single inhalation. N, sample size. A1: men’s symptoms following placebo administration; A2: women’s symptoms following placebo administration; B1: men’s symptoms following 500 mcg Δ^9^-THC administration; B2: women’s symptoms following 500 mcg Δ^9^-THC administration; C1: men’s symptoms following 1000 mcg Δ^9^-THC administration; C2: women’s symptoms following 1000 mcg Δ^9^-THC administration. Error bars represent standard deviation.

Our secondary analysis of the two PK studies found no significant differences between women and men in most of the common MC AEs or positively perceived effects (e.g., dizziness, tiredness, awareness or relaxation) at any time point (*p* > 0.05) (Figure 6).

## 3. Discussion

It is now widely accepted that women may process pain and respond to analgesics differently than men [20]. These differences are based on anatomical, psychological, neural, hormonal and cultural factors [21]. These differences were also demonstrated in male and female rodents that expressed different signaling pathways following injury [22,23]. Not much is known about the mechanism of cannabinoid analgesia and sex differences, but according to animal studies, males showed greater relief of symptoms in response to cannabinoids. Sex differences in cannabinoid metabolism might be associated with different reactions and receptor expressions and with hormonal differences [24]. Therefore, one would expect that chronic pain treatment in men and women should require different doses, result in different treatment responses and elicit different quantitative or qualitative AEs. Yet, this was not the case in the current study.

Here, no statistically significant sex differences were found in patients treated with MC via the Syqe MDI in short- or long-term effectiveness and safety, or in short-term Δ^9^-THC PK. As all patients were treated using the MDI, it allowed them to inhale precise, low doses of Δ^9^-THC, resulting in low inter-individual variability of blood concentration of Δ^9^-THC and its metabolites. While the mechanism of the clinical effects of Δ^9^-THC metabolites 11-hydroxy-Δ^9^-THC and Δ^9^-carboxy-THC are not yet fully understood, literature reviews on this issue humans are lacking and some in vivo studies suggested no to weak biological effects [25]. Nonetheless, another human study suggested they are highly active [26]. The high Δ^9^-THC doses of the other routes produce high Δ^9^-THC plasma levels (C_max_ range of ~50–250 ng/mL [27] and even up to 350 ng/mL [15]), much higher than those required for achieving pain relief here, and possibly resulting in a higher rate of AEs [28] as Wallace et al. (2020) showed in their study that the therapeutic window for optimal pain reduction is 16–31 ng/mL for plasma Δ^9^-THC [29].

Our analysis shows that at the endpoint of efficacy analysis (240 days of treatment), patients reported significant pain reduction with a mean of 1.58 points, or 2.60 points following imputation of missing data, without any difference between men and women other than at the D_14_ time point. The percentage of reduction in pain intensity (27%) was comparable or even somewhat better (23%) following 240 days of sublingual/smoked/vaped MC [18].

Although 26% of the patients in the current study reported no decrease in pain intensity at the end of the titration phase and 5% reported worsening of pain, these patients elected to continue using the inhaler, possibly as almost half of these patients experienced an improvement in sleep characteristics. Sleep measurements were found to be improved to a similar degree for both sexes. A previous study on MC and sleep found similar results [18] and it has even been argued that the effect of MC on patients’ wellness is related more to satisfactory sleep quality and quantity than to the improvement in pain intensity [30].

Notably, there were no differences between the sexes in AE rates. The rate of AEs during the study was low (10%), and most were reported during the titration phase, essentially disappearing following the attainment of a stable treatment regimen. In our previous studies [6,31], as well as in the United Kingdom Medical Cannabis Registry data [32], women reported higher AE rates than men, but they were also consuming different MC chemovars that contained different concentrations of phytocannabinoids and terpenoids. Therefore, it is not possible to distinguish between the effect of sex and the effect of MC variability. Since in the current study all patients were treated with the MDI and consumed only one MC cultivar, MC variability was controlled, possibly explaining why men and women reported the same low rate of AEs. Hence, we suggest that there are no sex-related differences in MC treatment and that the observed differences in previous studies are mainly due to the heterogeneity of MC treatment.

Of note, in the current study and in our previous one [6], although men consumed an absolute higher dose/amount of Δ^9^-THC /cannabis compared to females, the weight-adjusted dose was the same for both sexes. This is interesting with regard to physicians who do not think of dosing based on weight and the current study results suggest that physicians should pay more attention to this aspect in medical cannabis treatment.

In addition, contrary to previous studies on MC for chronic pain treatment in which MC quantities increased over time [18], we found that once stabilized, no increase in dose was required over a prolonged period in both men and women. 

Sex differences in MC treatment may be prevalent in aspects that were not assessed in the current study. Cuttler et al. (2016) reported that men used cannabis more frequently than women and more for recreational purposes, while women used cannabis more for medical indications [33]. Cannabis use in men led to higher rates of feeling hungry than in women, while the latter reported significantly more appetite loss. Men reported higher rates of altered sense of time, and were more enthusiastic than women.

### Limitations

This study has several limitations. First, like in most clinical studies on pain, the analyses relied on an NPS, which is a subjective tool for assessing pain intensity. Moreover, since women often experience and communicate pain differently than men, this and other systemic biases have to be acknowledged. Second, due to the ongoing nature of the study design and the 47% total attrition rate throughout the years of data collection, the sample size became smaller at each time point. This might have impacted the statistical results due to an increase in variability. Third, due to the heterogeneity of the sample, some of the treatment etiologies and the previous experience with MC measures varied significantly between the sexes. Fourth, this was an open-label study, with no randomization and with no placebo arm. Thus, it could be argued that some of the results were due to a placebo effect. Fifth, the sample size of the female groups for the PK studies is relatively small (n = 3–6); this calls into question the validity of sex comparisons for this measure. Nonetheless, this is the most comprehensive data currently available in the literature. Fifth, the MDI controls solely for Δ^9^-THC dose in the aerosol and not for the rest of the cannabis components that are emitted in the aerosol. Nonetheless, using one unique cannabis cultivar and the metered doses that are possible via the MDI should at least indicate some consistency of the other components. Sixth, due to the heterogeneity of the data relating to concomitant medications use, no sub-analyses were performed to assess the possible confounding effects.

## 4. Materials and Methods

### 4.1. General Considerations

Three forms of MC consumption are approved in Israel: inflorescences (for smoking or vaporizing administration), oil extracts (for sublingual use) and the Syqe MDI. The latter can be used only by patients for whom the use of Δ^9^-THC-rich MC was approved by the Israeli Ministry of Health (IMOH). Most licenses are issued for CNCP, preferably of neuropathic origin [34]. There are no additional exclusion criteria for the MDI treatment. 

### 4.2. Device

The Syqe MDI 1.1 is configured to use a vapor chip (VC) that delivers an aerosol containing either 250, 500, 750 or 1000 mcg Δ^9^-THC. Δ^9^-THC serves as an indicator for other phytocannabinoids, terpenoids and additional molecules in the whole inflorescence that are aerosolized concomitantly with it. Comprehensive information on the MDI and its use can be found in our previous publications [16,17,35,36]. The major phytocannabinoid concentrations of the unique medical cannabis cultivar used in the device (i.e., “Bedrocan” (Bedrocan International, Veendam, Netherlands)) have been previously published [36] (Figure S5). Specifically, these major phytocannabinoids in the plant are (−)-∆^9^-trans-tetrahydrocannabinol acid (THCA), THC, cannabigerolic acid (CBGA), cannabidiol acid (CBDA) and cannabinol (CBN). 

Syqe Medical provides all patients who use its MDI with a free patient support program (PSP). Upon joining this program, the patients provide informed consent, which allows data collection by the company’s PSP nursing team. In this study we retrospectively analyzed the data of all patients who were enrolled in the program between September 2019 and January 2023. Analysis of the collected data was approved by the Technion—Israel Institute of Technology’s Ethics Committee (#125-2021). The PSP also includes a call center that archives every AE report. 

This study was purely observational, using anonymized data, and not interventional (physicians prescribed medical cannabis via the MDI to patients due to their own clinical discretion so no official clinical trial registration was required).

### 4.3. Cohort Study

Treatment regimen: Once approved to use the Syqe MDI, a predefined amount of MDI cartridges per month was prescribed by the treating physician to each patient along with an individualized treatment regimen (i.e., number of inhalations and a titration plan).

Dose titration for most patients began with a dose of 250 mcg Δ^9^-THC twice per day. Thereafter, patients could add small incremental doses in accordance with their titration plan, which was subjected to either the absence of AEs for three consecutive days, or the presence of tolerable AEs, defined as AEs perceived by the patient as not preventing him/her from continuing the treatment (e.g., dry mouth, mild cough). Dose titration was supported and monitored by a designated PSP nurse, who assisted the patient in reaching a stable treatment regimen with as few AEs as possible. 

#### 4.3.1. Outcome Measures

Average weekly pain intensity was measured by a numerical pain scale (NPS) ranging from 0 (“no pain”) to 10 (“worst imaginable pain intensity”). Sleep timing (latency and duration) and quality were assessed by the Pittsburgh Sleep Quality Index (PSQI) [37]. Daily Δ^9^-THC dose was calculated at each follow-up time point based on the patient’s treatment regimen (Δ^9^-THC dose X number of inhalations per day). AEs were actively assessed by an open question: “Have you experienced any AE since the last follow-up?” at each follow-up visit. AEs were also recorded by the call center if a patient called to report such events spontaneously. AEs were categorized according to the MedDRA system organ class (SOC) for systems classification and by preferred term (PT) for the specific AEs.

Patient attrition from the program was recorded at predefined times: 1, 7, 14, 21, 30, 60, 90, 120, 180, 240, 360, 540, 720, 900, 1080 and 1260 days following treatment initiation. The reasons for attrition were also documented during the same call and assessed by an open question.

#### 4.3.2. Data Collection

Data were collected by the company’s PSP nurses based on outcomes reported by the patients. A baseline meeting during which the nurse instructed the patient on using the inhaler was held either in person at the patient’s home, by zoom video meeting or by a phone call. In this meeting, the nurse interviewed the patient about previous MC treatments, co-morbidities and concomitant medication use. Women were asked if they were pregnant or breastfeeding. Patient demographics like age, sex and body mass index (BMI) were also recorded. 

Inasmuch as possible, the same PSP nurse collected data on pain intensity and AEs by phone at predefined times as described above. At each call, women were also asked again if they were pregnant or breastfeeding. Patients were asked if they were continuing to use the MDI and if not, what the reason for stopping was. Patients were instructed to call the PSP support service if technical issues arise.

In this study, we reanalyzed the previously published, prospectively collected data of all patients who were enrolled in the program between September 2019 and October 2020 [19] with additional data collected up to January 2023.

### 4.4. PK Studies Design and Setting

The first study assessed the PK, safety and analgesic effect of a single 1000 mcg inhalation dose via Syqe’s MDI [17], conducted during November to December 2012. In this study, only the PK of Δ^9^-THC was evaluated, and no efficacy data were collected. The second MDI PK study was a randomized, 3-arm, double-blind, placebo-controlled, cross-over trial [16], conducted between March 2016 and July 2017. It assessed the differences in PK, safety and analgesic responses between 500 mcg Δ^9^-THC single inhalation per visit, 1000 mcg Δ^9^-THC single inhalation per visit and placebo (VC that does not contain Δ^9^-THC). In that study, we assessed the pharmacokinetics of Δ^9^-THC and its metabolites Δ^9^-carboxy-THC and 11-OH-Δ^9^-THC, in addition to the presence of symptoms that were scaled on a continuous rating (0–10 scale) of both frequently reported cannabis-related AEs (such as dizziness, dry mouth, headache, intoxication (“stoned”) feeling, nausea, throat soreness and tiredness) and frequently encountered positively perceived effects (such as awareness level, general feeling and relaxation). No sex differences were evaluated in these previous publications. The studies were conducted in the Pain Research Unit of Rambam Health Care Campus (Haifa, Israel), following approval by the Rambam Health Care Campus ethics committee (RMB 0131-13). All participants provided written informed consent. Patients were enrolled in the study after meeting the following criteria: (1) aged 18 years or older; (2) suffering from neuropathic pain of any type for at least 3 months; (3) stable analgesic regimen for at least 60 days that included MC; (4) normal liver function (defined as aspartate aminotransferase less than 3 times the normal level), normal renal function (defined as a serum creatinine level <1.50 mg/dL) and normal hematocrit (37–52%); (5) negative pregnancy test (β human chorionic gonadotropin pregnancy test), when applicable; and (6) possessed a valid license from the Israeli Ministry of Health to receive MC. Exclusion criteria were the presence of significant cardiac or pulmonary disease, history of a psychotic disorder, pregnancy or breastfeeding, or presence of non-neuropathic pain.

Notably, patients in those two PK studies were not cannabis naïve but were requested to refrain from consuming MC for at least 12 h prior to using the MDI. The washout period duration was based on findings reporting Δ^9^-THC elimination 30–60 min following cannabis smoking [27].

### 4.5. Statistical Analyses

Only intention-to-treat (ITT) population analyses were performed for all patients who were treated with the inhaler and had data for D_0_ (baseline) and at least one report for any of the follow-up time points. Categorical variables are presented as numbers and percentages. Distribution was assessed by the Shapiro–Wilk test of normality. Data with non-normal distribution are presented as the median and interquartile range (IQR) and normally distributed data are presented as mean ± standard deviation (SD). R software (V.1.1.463) with arsenal [38], atable [39] and tidyverse [40] packages were used to analyze the differences between men and women in all measures by Wilson’s chi-square test and Pearson’s chi-square for categorical measures and linear model ANOVA and Kruskal–Wallis rank sum test for numeric measures. The R package arsenal used only linear model ANOVA for comparison between two groups or more for all numerical normally distributed data. The lme4 package [41] was used to assess the overall effect on the outcome measures between D0 and the endpoint. Observations with <5 patient reports were considered non-applicable for statistical analysis, but were presented descriptively. Differences were considered significant if the *p*-value was lower than 0.05. Due to the prospective nature of the data, sample sizes are different between time points. Thus, demographic data in the results refer to the baseline (D_0_) time point only. Last observation carried forward (LOCF) was performed for patients with missing data and was carried forward for all subsequent observation points for the average weekly pain intensity outcome measures as a sensitivity analysis.

## 5. Conclusions

In conclusion, uniform MC treatment via the Syqe MDI showed no sex differences in short-term effectiveness, safety and PK, nor in long-term effects under “real-life” conditions and with no differences in attrition rates. These findings provide valuable insights into the effectiveness and safety of MC treatment for both sexes and may inform clinical practice and policy-making in the field. As such, regulators of the MC markets need to consider that sex-based MC product selection is not supported by the published literature. Hence, clinicians should be guided to a more evidence-based approach for MC treatment.

## Figures and Tables

**Table 1:** Baseline demographics by sex.

	Women N = 607	Men N = 513	Overall N = 1120	Statistic (*p*-Value)
	Mean ± SD			
BMI	27.0 ± 10.5	27.0 ± 4.72	27.0 ± 8.3	0 (0.98) ^†^
Age	59.5 ± 19.2	60.7 ± 16.2	60.1 ± 17.9	1.14 (0.28) ^†^
Previous cannabis use				
	N (%)			
Completely cannabis naïve	111 (18)	97 (19)	208 (19)	1.39 (0.3) ^††^
Previous recreational use	8 (1)	3 (<1)	11 (<1)	
Previous MC use	317 (52)	287 (56)	604 (54)	
Previous use of inflorescence	116 (19)	144 (28)	260 (23)	12.52 (<0.001) ^††^
Previous use of oil	271 (44)	219 (43)	490 (44)	0.43 (0.51) ^††^
Previous edibles	2 (<1)	1 (<1)	3 (<1)	NA
Previous RSO	1 (<1)	0 (<1)	1 (<1)	NA
Previous Sativex	2 (<1)	4 (<1)	6 (<1)	NA
Previous other	3 (<1)	3 (<1)	6 (<1)	NA
Intention of using MC concomitantly with the MDI (Yes)	92 (16)	94 (20)	186 (18)	3.26 (0.2) ^††^
MDI and MC concomitant use whilst having previous MC experience (n = 604)	83 (28)	86 (32)	169 (30)	1.68 (0.43) ^††^
	Mean ± SD			
MC dose gr/month	19.6 ± 10.8	23.0 ± 13.1	21.2 ± 12.1	9.9 (<0.005) ^†^
Previous MC use duration (months)	17.13 ± 22.03	27.76 ± 34.44	22.3 ± 29.19	15.87 (<0.001) ^†^

^†^, linear model ANOVA; ^††^, Pearson’s chi test; NA, not applicable; MDI, metered-dose inhaler; BMI, body mass index; MC, medical cannabis; RSO, Rick Simpson Oil.

**Table 2:** Reported treatment-related AEs by SOC by sex.

Adverse Events by SOC	Proportion of Women (CI 95%) N = 666	Proportion of Men (CI 95%) N = 582	Overall Proportion (CI 95%) N = 1248	Wilson’s χ^2^ Test (*p*-Value)
Any (at least 1 AE)	9.76 (7.73–12.25)	10.31 (8.09–13.05)	10.02 (8.47–11.81)	0.05 (0.82)
Nervous System Disorders	5.26 (3.8–7.22)	6.01 (4.36–8.25)	5.61 (4.46–7.03)	0.21 (0.65)
Gastrointestinal Disorders	3.15 (2.07–4.77)	2.23 (1.31–3.78)	2.72 (1.96–3.78)	0.67 (0.41)
Psychiatric Disorders	1.2 (0.61–2.35)	2.06 (1.18–3.57)	1.6 (1.04–2.46)	0.96 (0.33)
Respiratory, Thoracic and Mediastinal Disorders	1.95 (1.14–3.31)	1.55 (0.82–2.91)	1.76 (1.17–2.65)	2.43 (0.12)
General Disorders and Administration Site Conditions	2.1 (1.26–3.5)	0.86 (0.37–2)	1.52 (0.98–2.37)	0.11 (0.74)
Cardiac Disorders	0.45 (0.15–1.32)	0.86 (0.37–2)	0.64 (0.33–1.26)	0.3 (0.58)
Musculoskeletal and Connective Tissue Disorders	0 (0–0.57)	0.17 (0.03–0.97)	0.08 (0.01–0.45)	0 (0.95)
Skin and Subcutaneous Tissue Disorders	0 (0–0.57)	0.17 (0.03–0.97)	0.08 (0.01–0.45)	0 (0.95)
Vascular Disorders	0 (0–0.57)	0.17 (0.03–0.97)	0.08 (0.01–0.45)	0 (0.95)

SOC, system organ class.

## Data Availability

Data is contained within the article and supplementary material.

## Supplementary Materials

The following supporting information can be downloaded at: https://www.mdpi.com/article/10.3390/ph16101426/s1. Table S1: Attrition rate during the study by sex; Table S2: Overall attrition rate during the study by sex and by time points; Table S3: Specific reasons for attrition during the study by sex and by time points; Table S4: Specific sample etiologies/diagnoses for the treatment; Table S5: Specific comorbidities diagnoses; Table S6: Reported treatment-related specific AEs by sex; Table S7: Descriptive sample etiologies; Figure S1: Δ^9^-THC and its metabolites’ pharmacokinetics; Figure S2: Individualized sex differences in Δ^9^-THC and its metabolites’ pharmacokinetics; Figure S3: Sex comparison of blood pressure following a single inhalation dose; Figure S4: Sex comparison of heart rate following a single inhalation dose; Figure S5: U-HPLC analysis of the Bedrocan MC inflorescence product; Results S1: Pain reduction response comparison by sex.
