# Supplementary material for: Evaluating Sex Differences in Efficacy, Safety and Pharmacokinetics in Patients Treated with Cannabis by a Metered-Dose Inhaler
Source: Pharmaceuticals (Basel). 2023 Oct 8;16(10):1426. doi: 10.3390/ph16101426 (PMC10610259; doi:10.3390/ph16101426)
Supplement: Supplementary file 1 [file pharmaceuticals-16-01426-s001.zip › Supplemental materials 24.7.23 JA clean.pdf]

Supplemental Materials:

Real-world evidence data:

Table S1: Attrition rate during the study by sex.

Table S2: Overall attrition rate during the study by sex and by time points.

Table S3: Specific reasons for attrition rate during the study by sex and by time points.

Table S4: Specific sample etiologies/diagnoses for the treatment.

Table S5: Specific comorbidities diagnoses.

Table S6: Patients reported treatment-related specific AEs by sex.

Results S1: Pain reduction response comparison by sex.

Pharmacokinetic studies data:

Table S7: Descriptive sample etiologies.

Figure S1: Δ9-THC and its metabolites pharmacokinetics.

Figure S2: Individualized sex differences in Δ9-THC and its metabolites pharmacokinetics.

Figure S3: Sex comparison of blood pressure following a single inhalation.

Figure S4: Sex comparison of heart rate following a single inhalation.

Table S1: Attrition rate during the study by sex

Attrition reason	Women n=607 N (%)	Women proportion (CI 95%)	Men n=513 N (%)	Men proportional (CI 95%)	Overall sample N=1120 N (%)	Overall proportion (CI 95%)	Wilson's X^2 test (p-value)
Total	433 (71)	71. 3 (CI 95%:67.6-74.8)	366 (71)	71. 3 (CI 95%:67.3-75.1)	799 (71)	71. 3 (CI 95%:67.7-74.6)	0 (1)
Ineffectiveness	122 (20)	20.1 (CI 95%:17.1-23.47)	97 (19)	18.91 (CI 95%:15.76-22.52)	219 (0)	19.55 (CI 95%:17.34-21.98)	0.18 (0.67)
Other reason	62 (10)	10.21 (CI 95%:8.05-12.88)	45 (9)	8.77 (CI 95%:6.62-11.54	107 (0)	9.55 (CI 95%:7.97-11.42)	0.51 (0.47)
Switched to other MC form	51 (8)	8.4 CI 95%:6.45-10.88)	63 (12)	12.28 (CI 95%:9.72-15.4)	114 (0)	10.18 (CI 95%:8.54-12.09)	4.16 (0.04)
Financial	35 (6)	5.77 (CI 95%:4.17-7.91)	31 (6)	6.04 (CI 95%:4.29-8.45)	66 (0)	5.89 (CI 95%:4.66-7.43)	0 (0.95)
Adverse Event	35 (6)	5.77 (CI 95%:4.17-7.91)	26 (5)	5.07 (CI 95%:3.48-7.32)	61 (0)	5.45 (CI 95%:4.26-6.93)	0.15 (0.7)
Switched to other non-MC treatment	27 (4)	4.45 CI 95%:3.07-6.39)	15 (3)	2.92 (CI 95%:1.78-4.77)	42 (0)	3.75 (CI 95%:2.79-5.03)	1.39 (0.24)
Physician decision permanently	23 (4)	3.79 (CI 95%:2.54-5.62)	24 (5)	4.68 (CI 95%:3.16-6.87	47 (0)	4.2 (CI 95%:3.17-5.54)	0.35 (0.56)
License expired	21 (3)	3.46 (CI 95%:2.27-5.23)	13 (3)	2.53 (CI 95%:1.49-4.29)	34 (0)	3.04 (CI 95%:2.18-4.21)	0.53 (0.47)
Improved health	19 (3)	3.13 (CI 95%:2.01-4.84)	14 (3)	2.73 (CI 95%:1.63-4.53)	33 (0)	2.95 (CI 95%:2.11-4.11)	0.05 (0.83)

Health Worsening	16 (3)	2.64 (CI 95%:1.63-4.24)	12 (2)	2.34 (CI 95%:1.34-4.04)	28 (0)	2.5 (CI 95%:1.74-3.59)	0.02 (0.9)
Deceased	11 (2)	1.81 (CI 95%:1.01-3.22)	17 (3)	3.31 (CI 95%:2.08-5.24)	28 (0)	2.5 (CI 95%:1.74-3.59)	1.99 (0.16)
Physician decision temporary	6 (1)	0.99 CI 95%:0.45-2.14)	5 (1)	0.97 (CI 95%:0.42-2.26)	11 (0)	0.98 (CI 95%:0.55-1.75)	0 (1)
Immigration	3 (0)	0.49 (CI 95%:0.17-1.44)	1 (0)	0.19 (CI 95%:0.03-1.1)	4 (0)	0.36 (CI 95%:0.14-0.91)	0.11 (0.74)
Intermittently continued	1 (0)	0.16 (CI 95%:0.03-0.93)	2 (0)	0.39 (CI 95%:0.11-1.41)	3 (0)	0.27 (CI 95%:0.09-0.78)	0.02 (0.88)
Sudden illness temp	1 (0)	0.16 CI 95%:0.03-0.93)	1 (0)	0.19 CI 95%:0.03-1.1)	2 (0)	0.18 (CI 95%:0.05-0.65)	0 (1)

MC, Medical cannabis; Numbers do not add up to 100% due to concomitant reasons.

Table S2: Overall attrition rate during the study by sex and by time points

Time point range (days)	Women n=607 N (%)	Women proportion (CI 95%)	Men n=513 N (%)	Men proportional (CI 95%)	Overall sample N=1120 N (%)	Overall proportion (CI 95%)	Wilson's X^2 test (p-value)
**D_0-D_1**	3 (0)	0.5 (CI 95%:0.17-1.45)	4 (1)	0.79 (CI 95%:0.31-2	7 (1)	0.63 (CI 95%:0.16-0.05)	0.05 (0.82)
**D_1-D_7**	5 (1)	0.83 ((CI 95%:0.36-1.94)	6 (1)	1.19 (CI 95%:0.55-2.58	11 (1)	1 (CI 95%:0.41-0.07)	0.09 (0.77)
**D_7-D_{14}**	10 (2)	1.7 (CI 95%:0.92-3.1	8 (2)	1.62 (CI 95%:0.82-3.16	18 (2)	1.66 (CI 95%:0.9-0.09)	0 (1)
**D_{14}-D_{21}**	18 (3)	3.15 (CI 95%:2-4.93	15 (3)	3.13 (CI 95%:1.9-5.09	33 (3)	3.14 (CI 95%:2.09-0.14)	0 (1)
**D_{21}-D_{30}**	30 (6)	5.55 (CI 95%:3.91-7.81	20 (4)	4.35 (CI 95%:2.83-6.62	50 (4)	5 (CI 95%:3.65-0.18)	0.52 (0.47)
**D_{30}-D_{60}**	61 (13)	12.71 (CI 95%:10.02-15.99	45 (11)	10.84 (CI 95%:8.2-14.2	106 (9)	11.84 (CI 95%:9.73-0.33)	0.57 (0.45)
**D_{60}-D_{90}**	35 (8)	7.87 (CI 95%:5.71-10.74	42 (11)	11.26 (CI 95%:8.44-14.87	77 (7)	9.41 (CI 95%:7.41-0.29)	2.36 (0.13)
**D_{90}-D_{120}**	25 (6)	5.95 (CI 95%:4.06-8.64	26 (7)	7.49 (CI 95%:5.16-10.75	51 (5)	6.65 (CI 95%:4.89-0.24)	0.5 (0.48)
**D_{120}-D_{180}**	43 (11)	11.41 (CI 95%:8.58-15.01	33 (11)	10.51 (CI 95%:7.58-14.39	76 (7)	11 (CI 95%:8.67-0.34)	0.06 (0.8)
**D_{180}-D_{240}**	18 (5)	5.01 (CI 95%:3.19-7.79	14 (5)	4.67 (CI 95%:2.8-7.68	32 (3)	4.86 (CI 95%:3.21-0.21)	0 (0.98)
**D_{240}-D_{360}**	13 (4)	3.76 (CI 95%:2.21-6.32	11 (4)	3.81 (CI 95%:2.14-6.69	24 (2)	3.78 (CI 95%:2.3-0.19)	0 (1)

**D_{360}-D_{540}**	11 (3)	3.28 (CI 95%:1.84-5.78	6 (2)	2.12 (CI 95%:0.98-4.55	17 (2)	2.75 (CI 95%:1.46-0.16)	0.4 (0.53)
**D_{540}-D_{720}**	3 (1)	0.9 (CI 95%:0.31-2.62	6 (2)	2.17 (CI 95%:1-4.64	9 (1)	1.48 (CI 95%:0.52-0.11)	NA
**D_{720}-D_{900}**	1 (0)	0.3 (CI 95%:0.05-1.69	2 (1)	0.73 (CI 95%:0.2-2.61	3 (0)	0.5 (CI 95%:0-0.06)	NA
**D_{900}-D_{1080}**	2 (1)	0.61 (CI 95%:0.17-2.19	3 (1)	1.1 (CI 95%:0.38-3.19	5 (0)	0.83 (CI 95%:0.11-0.08)	NA
**D_{1080}-D_{1260}**	2 (1)	0.61 (CI 95%:0.17-2.2	3 (1)	1.12 (CI 95%:0.38-3.23	5 (0)	0.84 (CI 95%:0.11-0.08)	NA

MC, Medical cannabis; Numbers do not add to 100% due to concomitant reasons; only time points with dropout reports are indicated.

Table S3: Specific reasons for attrition rates during the study by sex and by time points

Time period	Attrition Reason	Women n=607 N (%)	Women proportion (CI 95%)	Men n=513 N (%)	Men proportional (CI 95%)	Overall sample N=1120 N (%)	Overall proportion (CI 95%)	Wilson's X^2 test (p-value)
**D_0-D_1**	Adverse Event	2 (0)	0.33 CI 95%:0.09-1.2	1 (0)	0.2 CI 95%:0.03-1.1	3 (0)	0.27 (CI 95%:0-0.03)	NA
**D_0-D_1**	Other reason	1 (0)	0.17 CI 95%:0.03-0.93	1 (0)	0.2 CI 95%:0.03-1.1	2 (0)	0.18 (CI 95%:0-0.03)	NA
**D_0-D_1**	Ineffectiveness	0 (0)	0 CI 95%:0-0.63	1 (0)	0.2 CI 95%:0.03-1.1	1 (0)	0.09 (CI 95%:0-0.02)	NA
**D_0-D_1**	Physician decision temporarily	0 (0)	0 CI 95%:0-0.63	1 (0)	0.2 CI 95%:0.03-1.1	1 (0)	0.09 (CI 95%:0-0.02)	NA
**D_0-D_1**	Switched to other MC form	0 (0)	0 CI 95%:0-0.63	1 (0)	0.2 CI 95%:0.03-1.11	1 (0)	0.09 (CI 95%:0-0.02)	NA
**D_1-D_7**	Adverse Event	3 (0)	0.5 CI 95%:0.17-1.46	2 (0)	0.4 CI 95%:0.11-1.43	5 (0)	0.45 (CI 95%:0.03-0.04)	NA
**D_1-D_7**	Other reason	2 (0)	0.33 CI 95%:0.09-1.21	2 (0)	0.4 CI 95%:0.11-1.44	4 (0)	0.36 (CI 95%:0-0.04)	NA
**D_1-D_7**	Switched to other MC form	0 (0)	0 CI 95%:0-0.64	2 (0)	0.4 CI 95%:0.11-1.45	2 (0)	0.18 (CI 95%:0-0.03)	NA
**D_1-D_7**	Deceased	0 (0)	0 CI 95%:0-0.64	1 (0)	0.2 CI 95%:0.03-1.11	1 (0)	0.09 (CI 95%:0-0.02)	NA
**D_1-D_7**	Health Worsening	0 (0)	0 CI 95%:0-0.64	1 (0)	0.2 CI 95%:0.04-1.12	1 (0)	0.09 (CI 95%:0-0.02)	NA
**D_1-D_7**	Ineffectiveness	0 (0)	0 CI 95%:0-0.64	1 (0)	0.2 CI 95%:0.04-1.12	1 (0)	0.09 (CI 95%:0-0.02)	NA
**D_7-D_{14}**	Other reason	3 (1)	0.51 CI 95%:0.17-1.48	4 (1)	0.82 CI 95%:0.32-2.09	7 (1)	0.65 (CI 95%:0.03-0.05)	NA

**D_7-D_{14}**	Physician decision permanently	4 (1)	0.68 CI 95%:0.27-1.74	0 (0)	0 CI 95%:0-0.78	4 (0)	0.37 (CI 95%:0.1-0.04)	NA
**D_7-D_{14}**	Ineffectiveness	1 (0)	0.17 CI 95%:0.03-0.95	4 (1)	0.81 CI 95%:0.32-2.07	5 (0)	0.46 (CI 95%:0-0.04)	NA
**D_7-D_{14}**	Switched to other MC form	2 (0)	0.34 CI 95%:0.09-1.24	3 (1)	0.62 CI 95%:0.21-1.8	5 (0)	0.47 (CI 95%:0-0.05)	NA
**D_7-D_{14}**	Adverse Event	3 (1)	0.5 CI 95%:0.17-1.47	1 (0)	0.2 CI 95%:0.04-1.13	4 (0)	0.37 (CI 95%:0.03-0.04)	NA
**D_7-D_{14}**	Deceased	1 (0)	0.17 CI 95%:0.03-0.95	1 (0)	0.2 CI 95%:0.04-1.13	2 (0)	0.18 (CI 95%:0-0.03)	NA
**D_7-D_{14}**	Improved health	1 (0)	0.17 CI 95%:0.03-0.95	1 (0)	0.2 CI 95%:0.04-1.13	2 (0)	0.18 (CI 95%:0-0.03)	NA
**D_{14}-D_{21}**	Ineffectiveness	10 (2)	1.77 CI 95%:0.96-3.22	6 (1)	1.28 CI 95%:0.59-2.76	16 (2)	1.54 (CI 95%:0.59-0.09)	0.15 (0.7)
**D_{14}-D_{21}**	Adverse Event	6 (1)	1.04 CI 95%:0.48-2.25	7 (1)	1.46 CI 95%:0.71-2.99	13 (1)	1.23 (CI 95%:0.25-0.08)	0.12 (0.73)
**D_{14}-D_{21}**	Switched to other MC form	6 (1)	1.08 CI 95%:0.5-2.34	5 (1)	1.09 CI 95%:0.47-2.54	11 (1)	1.09 (CI 95%:0.27-0.07)	0 (1)
**D_{14}-D_{21}**	Other reason	5 (1)	0.89 CI 95%:0.38-2.07	5 (1)	1.08 CI 95%:0.46-2.49	10 (1)	0.97 (CI 95%:0.18-0.07)	0 (1)
**D_{14}-D_{21}**	Switched to other non-MC treatment	4 (1)	0.73 CI 95%:0.28-1.85	0 (0)	0 CI 95%:0-0.83	4 (0)	0.4 (CI 95%:0.11-0.04)	NA
**D_{14}-D_{21}**	Financial	2 (0)	0.35 CI 95%:0.1-1.26	1 (0)	0.21 CI 95%:0.04-1.18	3 (0)	0.29 (CI 95%:0-0.04)	NA
**D_{14}-D_{21}**	Physician decision permanently	1 (0)	0.18 CI 95%:0.03-1	2 (0)	0.43 CI 95%:0.12-1.56	3 (0)	0.29 (CI 95%:0-0.04)	NA
**D_{14}-D_{21}**	Physician decision temporarily	0 (0)	0 CI 95%:0-0.68	1 (0)	0.22 CI 95%:0.04-1.22	1 (0)	0.1 (CI 95%:0-0.02)	NA
**D_{14}-D_{21}**	Deceased	0 (0)	0 CI 95%:0-0.66	1 (0)	0.21 CI 95%:0.04-1.18	1 (0)	0.09 (CI 95%:0-0.02)	NA
**D_{21}-D_{30}**	Ineffectiveness	14 (3)	2.67 CI 95%:1.6-4.43	10 (2)	2.28 CI 95%:1.24-4.14	24 (2)	2.49 (CI 95%:1.05-0.12)	0.03 (0.86)

**D_{21}-D_{30}**	Other reason	11 (2)	2.14 CI 95%:1.2-3.79	5 (1)	1.16 CI 95%:0.5-2.68	16 (2)	1.69 (CI 95%:0.77-0.1)	0.84 (0.36)
**D_{21}-D_{30}**	Financial	6 (1)	1.11 CI 95%:0.51-2.4	5 (1)	1.11 CI 95%:0.47-2.57	11 (1)	1.11 (CI 95%:0.28-0.08)	0 (1)
**D_{21}-D_{30}**	Switched to other MC form	4 (1)	0.79 CI 95%:0.31-2	4 (1)	0.94 CI 95%:0.36-2.38	8 (1)	0.85 (CI 95%:0.13-0.07)	NA
**D_{21}-D_{30}**	Adverse Event	4 (1)	0.73 CI 95%:0.29-1.87	1 (0)	0.22 CI 95%:0.04-1.23	5 (0)	0.5 (CI 95%:0.11-0.05)	NA
**D_{21}-D_{30}**	Switched to other non-MC treatment	2 (0)	0.39 CI 95%:0.11-1.43	1 (0)	0.23 CI 95%:0.04-1.32	3 (0)	0.32 (CI 95%:0-0.04)	NA
**D_{21}-D_{30}**	Health Worsening	1 (0)	0.19 CI 95%:0.03-1.04	2 (0)	0.45 CI 95%:0.12-1.61	3 (0)	0.3 (CI 95%:0-0.04)	NA
**D_{21}-D_{30}**	Physician decision permanently	1 (0)	0.19 CI 95%:0.03-1.1	1 (0)	0.23 CI 95%:0.04-1.3	2 (0)	0.21 (CI 95%:0-0.03)	NA
**D_{21}-D_{30}**	License expired	0 (0)	0 CI 95%:0-0.73	2 (0)	0.46 CI 95%:0.13-1.65	2 (0)	0.21 (CI 95%:0-0.03)	NA
**D_{30}-D_{60}**	Ineffectiveness	35 (8)	8.05 CI 95%:5.84-10.98	21 (5)	5.48 CI 95%:3.61-8.24	56 (7)	6.85 (CI 95%:4.04-0.24)	1.72 (0.19)
**D_{30}-D_{60}**	Switched to other MC form	12 (3)	2.93 CI 95%:1.68-5.05	12 (3)	3.35 CI 95%:1.93-5.77	24 (3)	3.12 (CI 95%:1.18-0.15)	0.02 (0.9)
**D_{30}-D_{60}**	Financial	13 (3)	2.71 CI 95%:1.59-4.59	11 (3)	2.71 CI 95%:1.52-4.79	24 (3)	2.71 (CI 95%:1.07-0.13)	0 (1)
**D_{30}-D_{60}**	Other reason	10 (2)	2.35 CI 95%:1.28-4.28	7 (2)	1.86 CI 95%:0.9-3.79	17 (2)	2.12 (CI 95%:0.87-0.12)	0.06 (0.81)
**D_{30}-D_{60}**	Adverse Event	11 (2)	2.22 CI 95%:1.24-3.93	6 (1)	1.43 CI 95%:0.66-3.08	17 (2)	1.86 (CI 95%:0.81-0.11)	0.41 (0.53)
**D_{30}-D_{60}**	Health Worsening	6 (1)	1.27 CI 95%:0.58-2.74	2 (0)	0.5 CI 95%:0.14-1.79	8 (1)	0.91 (CI 95%:0.34-0.07)	NA
**D_{30}-D_{60}**	Physician decision permanently	3 (1)	0.71 CI 95%:0.24-2.07	5 (1)	1.35 CI 95%:0.58-3.12	8 (1)	1.01 (CI 95%:0.08-0.08)	NA
**D_{30}-D_{60}**	Deceased	4 (1)	0.81 CI 95%:0.32-2.07	3 (1)	0.72 CI 95%:0.24-2.09	7 (1)	0.77 (CI 95%:0.14-0.06)	NA

**D_{30}-D_{60}**	Switched to other non-MC treatment	3 (1)	0.74 CI 95%:0.25-2.14	2 (1)	0.56 CI 95%:0.15-2.02	5 (1)	0.66 (CI 95%:0.09-0.06)	NA
**D_{30}-D_{60}**	Improved health	2 (0)	0.43 CI 95%:0.12-1.54	0 (0)	0 CI 95%:0-0.94	2 (0)	0.23 (CI 95%:0-0.03)	NA
**D_{30}-D_{60}**	Physician decision temporarily	0 (0)	0 CI 95%:0-0.9	1 (0)	0.27 CI 95%:0.05-1.51	1 (0)	0.13 (CI 95%:0-0.03)	NA
**D_{30}-D_{60}**	Immigration	1 (0)	0.21 CI 95%:0.04-1.19	0 (0)	0 CI 95%:0-0.94	1 (0)	0.11 (CI 95%:0-0.02)	NA
**D_{60}-D_{90}**	Ineffectiveness	19 (5)	5.04 CI 95%:3.25-7.74	19 (6)	5.94 CI 95%:3.83-9.09	38 (5)	5.45 (CI 95%:2.41-0.22)	0.12 (0.72)
**D_{60}-D_{90}**	Switched to other MC form	6 (2)	1.68 CI 95%:0.77-3.62	12 (4)	4.1 CI 95%:2.36-7.02	18 (3)	2.77 (CI 95%:0.55-0.15)	2.65 (0.1)
**D_{60}-D_{90}**	Other reason	8 (2)	2.19 CI 95%:1.11-4.25	8 (3)	2.58 CI 95%:1.31-5.01	16 (2)	2.37 (CI 95%:0.81-0.14)	0.01 (0.93)
**D_{60}-D_{90}**	Financial	3 (1)	0.75 CI 95%:0.26-2.18	7 (2)	2.06 CI 95%:1-4.19	10 (1)	1.35 (CI 95%:0.1-0.1)	NA
**D_{60}-D_{90}**	Adverse Event	3 (1)	0.74 CI 95%:0.25-2.16	6 (2)	1.71 CI 95%:0.79-3.69	9 (1)	1.19 (CI 95%:0.1-0.09)	NA
**D_{60}-D_{90}**	Physician decision permanently	3 (1)	0.83 CI 95%:0.28-2.4	4 (1)	1.31 CI 95%:0.51-3.31	7 (1)	1.05 (CI 95%:0.13-0.09)	NA
**D_{60}-D_{90}**	Switched to other non-MC treatment	3 (1)	0.85 CI 95%:0.29-2.46	3 (1)	1.03 CI 95%:0.35-3	6 (1)	0.93 (CI 95%:0.14-0.08)	NA
**D_{60}-D_{90}**	License expired	3 (1)	0.8 CI 95%:0.27-2.33	2 (1)	0.63 CI 95%:0.17-2.26	5 (1)	0.72 (CI 95%:0.12-0.07)	NA
**D_{60}-D_{90}**	Deceased	1 (0)	0.25 CI 95%:0.04-1.39	3 (1)	0.86 CI 95%:0.29-2.51	4 (1)	0.53 (CI 95%:0-0.06)	NA
**D_{60}-D_{90}**	Improved health	2 (1)	0.51 CI 95%:0.14-1.82	1 (0)	0.29 CI 95%:0.05-1.65	3 (0)	0.41 (CI 95%:0-0.05)	NA
**D_{60}-D_{90}**	Health Worsening	2 (1)	0.5 CI 95%:0.14-1.81	0 (0)	0 CI 95%:0-1.12	2 (0)	0.27 (CI 95%:0-0.04)	NA
**D_{60}-D_{90}**	Physician decision temporarily	0 (0)	0 CI 95%:0-1.05	1 (0)	0.33 CI 95%:0.06-1.83	1 (0)	0.15 (CI 95%:0-0.03)	NA

**D_{90}-D_{120}**	Ineffectiveness	12 (4)	3.59 CI 95%:2.07-6.17	12 (4)	4.48 CI 95%:2.58-7.66	24 (4)	3.99 (CI 95%:1.65-0.2)	0.12 (0.73)
**D_{90}-D_{120}**	Other reason	6 (2)	1.83 CI 95%:0.84-3.93	7 (3)	2.69 CI 95%:1.31-5.45	13 (2)	2.21 (CI 95%:0.65-0.14)	0.18 (0.67)
**D_{90}-D_{120}**	Switched to other MC form	3 (1)	0.93 CI 95%:0.32-2.71	7 (3)	2.79 CI 95%:1.36-5.64	10 (2)	1.75 (CI 95%:0.18-0.12)	NA
**D_{90}-D_{120}**	Financial	3 (1)	0.86 CI 95%:0.29-2.5	4 (1)	1.42 CI 95%:0.55-3.59	7 (1)	1.11 (CI 95%:0.15-0.09)	NA
**D_{90}-D_{120}**	Switched to other non-MC treatment	3 (1)	0.94 CI 95%:0.32-2.74	3 (1)	1.21 CI 95%:0.41-3.5	6 (1)	1.06 (CI 95%:0.19-0.09)	NA
**D_{90}-D_{120}**	Deceased	2 (1)	0.57 CI 95%:0.16-2.05	3 (1)	1.05 CI 95%:0.36-3.04	5 (1)	0.78 (CI 95%:0.02-0.08)	NA
**D_{90}-D_{120}**	Physician decision permanently	2 (1)	0.61 CI 95%:0.17-2.21	2 (1)	0.78 CI 95%:0.21-2.78	4 (1)	0.68 (CI 95%:0.03-0.07)	NA
**D_{90}-D_{120}**	Physician decision temporarily	2 (1)	0.62 CI 95%:0.17-2.22	0 (0)	0 CI 95%:0-1.47	2 (0)	0.34 (CI 95%:0.04-0.05)	NA
**D_{90}-D_{120}**	Health Worsening	1 (0)	0.29 CI 95%:0.05-1.61	1 (0)	0.36 CI 95%:0.06-1.99	2 (0)	0.32 (CI 95%:0-0.05)	NA
**D_{90}-D_{120}**	Adverse Event	1 (0)	0.28 CI 95%:0.05-1.59	1 (0)	0.35 CI 95%:0.06-1.93	2 (0)	0.31 (CI 95%:0-0.05)	NA
**D_{90}-D_{120}**	Intermittently continued	0 (0)	0 CI 95%:0-1.14	1 (0)	0.37 CI 95%:0.07-2.09	1 (0)	0.17 (CI 95%:0-0.03)	NA
**D_{90}-D_{120}**	Immigration	1 (0)	0.29 CI 95%:0.05-1.62	0 (0)	0 CI 95%:0-1.35	1 (0)	0.16 (CI 95%:0-0.03)	NA
**D_{90}-D_{120}**	Improved health	0 (0)	0 CI 95%:0-1.1	1 (0)	0.36 CI 95%:0.06-1.99	1 (0)	0.16 (CI 95%:0-0.03)	NA
**D_{120}-D_{180}**	Ineffectiveness	16 (5)	5.48 CI 95%:3.4-8.71	12 (5)	5.31 CI 95%:3.06-9.05	28 (5)	5.41 (CI 95%:2.93-0.25)	0 (1)
**D_{120}-D_{180}**	Other reason	11 (4)	3.97 CI 95%:2.23-6.97	3 (1)	1.37 CI 95%:0.47-3.95	14 (3)	2.82 (CI 95%:1.98-0.17)	2.14 (0.14)
**D_{120}-D_{180}**	Switched to other MC form	7 (3)	2.66 CI 95%:1.3-5.39	9 (4)	4.39 CI 95%:2.33-8.13	16 (3)	3.42 (CI 95%:1.19-0.2)	0.59 (0.44)

**D_{120}-D_{180}**	Physician decision permanently	5 (2)	1.84 CI 95%:0.79-4.23	5 (2)	2.34 CI 95%:1-5.35	10 (2)	2.06 (CI 95%:0.69-0.15)	0 (0.95)
**D_{120}-D_{180}**	Switched to other non-MC treatment	5 (2)	1.94 CI 95%:0.83-4.46	2 (1)	0.99 CI 95%:0.27-3.52	7 (2)	1.52 (CI 95%:0.76-0.13)	NA
**D_{120}-D_{180}**	Improved health	5 (2)	1.62 CI 95%:0.7-3.74	4 (2)	1.68 CI 95%:0.66-4.24	9 (2)	1.65 (CI 95%:0.55-0.12)	NA
**D_{120}-D_{180}**	License expired	4 (1)	1.39 CI 95%:0.54-3.52	3 (1)	1.35 CI 95%:0.46-3.9	7 (1)	1.37 (CI 95%:0.43-0.11)	NA
**D_{120}-D_{180}**	Deceased	2 (1)	0.63 CI 95%:0.17-2.28	2 (1)	0.82 CI 95%:0.22-2.93	4 (1)	0.71 (CI 95%:0.04-0.08)	NA
**D_{120}-D_{180}**	Health Worsening	2 (1)	0.64 CI 95%:0.18-2.3	1 (0)	0.41 CI 95%:0.07-2.3	3 (1)	0.54 (CI 95%:0.05-0.07)	NA
**D_{120}-D_{180}**	Financial	1 (0)	0.32 CI 95%:0.06-1.78	2 (1)	0.82 CI 95%:0.23-2.95	3 (1)	0.54 (CI 95%:0-0.07)	NA
**D_{120}-D_{180}**	Physician decision temporarily	2 (1)	0.74 CI 95%:0.2-2.66	0 (0)	0 CI 95%:0-1.76	2 (0)	0.41 (CI 95%:0.09-0.06)	NA
**D_{120}-D_{180}**	Intermittently continued	0 (0)	0 CI 95%:0-1.3	1 (0)	0.44 CI 95%:0.08-2.47	1 (0)	0.19 (CI 95%:0-0.04)	NA
**D_{120}-D_{180}**	Adverse Event	0 (0)	0 CI 95%:0-1.19	1 (0)	0.4 CI 95%:0.07-2.26	1 (0)	0.18 (CI 95%:0-0.04)	NA
**D_{180}-D_{240}**	Ineffectiveness	6 (2)	2.43 CI 95%:1.12-5.2	5 (3)	2.56 CI 95%:1.1-5.86	11 (2)	2.49 (CI 95%:1.07-0.17)	0 (1)
**D_{180}-D_{240}**	Switched to other MC form	6 (3)	2.55 CI 95%:1.18-5.46	2 (1)	1.07 CI 95%:0.29-3.82	8 (2)	1.9 (CI 95%:1.16-0.15)	NA
**D_{180}-D_{240}**	License expired	4 (2)	1.65 CI 95%:0.64-4.16	1 (1)	0.52 CI 95%:0.09-2.86	5 (1)	1.14 (CI 95%:0.6-0.11)	NA
**D_{180}-D_{240}**	Switched to other non-MC treatment	2 (1)	0.86 CI 95%:0.24-3.08	3 (2)	1.63 CI 95%:0.56-4.68	5 (1)	1.2 (CI 95%:0.17-0.12)	NA
**D_{180}-D_{240}**	Adverse Event	2 (1)	0.78 CI 95%:0.21-2.8	0 (0)	0 CI 95%:0-1.86	2 (0)	0.44 (CI 95%:0.12-0.06)	NA
**D_{180}-D_{240}**	Financial	2 (1)	0.79 CI 95%:0.22-2.82	0 (0)	0 CI 95%:0-1.87	2 (0)	0.44 (CI 95%:0.12-0.07)	NA

**D_{180}-D_{240}**	Sudden illness temp	1 (0)	0.41 CI 95%:0.07-2.31	1 (1)	0.53 CI 95%:0.09-2.94	2 (0)	0.47 (CI 95%:0-0.07)	NA
**D_{180}-D_{240}**	Physician decision permanently	1 (0)	0.41 CI 95%:0.07-2.3	1 (1)	0.52 CI 95%:0.09-2.91	2 (0)	0.46 (CI 95%:0-0.07)	NA
**D_{180}-D_{240}**	Other reason	0 (0)	0 CI 95%:0-1.56	2 (1)	1.04 CI 95%:0.29-3.72	2 (0)	0.46 (CI 95%:0-0.07)	NA
**D_{180}-D_{240}**	Improved health	1 (0)	0.4 CI 95%:0.07-2.2	1 (1)	0.5 CI 95%:0.09-2.78	2 (0)	0.44 (CI 95%:0-0.07)	NA
**D_{180}-D_{240}**	Deceased	0 (0)	0 CI 95%:0-1.48	1 (0)	0.5 CI 95%:0.09-2.75	1 (0)	0.22 (CI 95%:0-0.04)	NA
**D_{180}-D_{240}**	Physician decision temporarily	0 (0)	0 CI 95%:0-1.56	1 (1)	0.53 CI 95%:0.09-2.92	1 (0)	0.23 (CI 95%:0-0.05)	NA
**D_{180}-D_{240}**	Health Worsening	0 (0)	0 CI 95%:0-1.49	1 (0)	0.5 CI 95%:0.09-2.76	1 (0)	0.22 (CI 95%:0-0.05)	NA
**D_{240}-D_{360}**	License expired	7 (3)	3.21 CI 95%:1.56-6.48	1 (1)	0.56 CI 95%:0.1-3.11	8 (2)	2.02 (CI 95%:1.62-0.16)	NA
**D_{240}-D_{360}**	Switched to other MC form	3 (1)	1.43 CI 95%:0.49-4.12	6 (4)	3.51 CI 95%:1.62-7.44	9 (2)	2.36 (CI 95%:0.51-0.18)	NA
**D_{240}-D_{360}**	Ineffectiveness	4 (2)	1.78 CI 95%:0.69-4.48	4 (2)	2.23 CI 95%:0.87-5.6	8 (2)	1.98 (CI 95%:0.7-0.16)	NA
**D_{240}-D_{360}**	Other reason	3 (1)	1.4 CI 95%:0.48-4.02	1 (1)	0.56 CI 95%:0.1-3.13	4 (1)	1.02 (CI 95%:0.48-0.11)	NA
**D_{240}-D_{360}**	Switched to other non-MC treatment	3 (1)	1.45 CI 95%:0.49-4.17	0 (0)	0 CI 95%:0-2.2	3 (1)	0.79 (CI 95%:0.52-0.1)	NA
**D_{240}-D_{360}**	Improved health	2 (1)	0.87 CI 95%:0.24-3.13	0 (0)	0 CI 95%:0-2.06	2 (0)	0.49 (CI 95%:0.17-0.07)	NA
**D_{240}-D_{360}**	Financial	1 (0)	0.43 CI 95%:0.08-2.4	1 (1)	0.55 CI 95%:0.1-3.03	2 (0)	0.48 (CI 95%:0-0.07)	NA

**D_{240}-D_{360}**	Physician decision permanently	1 (0)	0.47 CI 95%:0.08-2.6	0 (0)	0 CI 95%:0-2.12	1 (0)	0.26 (CI 95%:0-0.05)	NA
**D_{240}-D_{360}**	Physician decision temporarily	1 (0)	0.47 CI 95%:0.08-2.61	0 (0)	0 CI 95%:0-2.12	1 (0)	0.26 (CI 95%:0-0.05)	NA
**D_{240}-D_{360}**	Immigration	1 (0)	0.43 CI 95%:0.08-2.41	0 (0)	0 CI 95%:0-2.06	1 (0)	0.24 (CI 95%:0-0.05)	NA
**D_{360}-D_{540}**	Ineffectiveness	4 (2)	2.04 CI 95%:0.8-5.13	2 (1)	1.2 CI 95%:0.33-4.29	6 (2)	1.66 (CI 95%:0.91-0.15)	NA
**D_{360}-D_{540}**	Financial	3 (1)	1.48 CI 95%:0.5-4.25	0 (0)	0 CI 95%:0-2.21	3 (1)	0.8 (CI 95%:0.54-0.1)	NA
**D_{360}-D_{540}**	License expaired	2 (1)	1.03 CI 95%:0.28-3.68	1 (1)	0.61 CI 95%:0.11-3.35	3 (1)	0.84 (CI 95%:0.29-0.1)	NA
**D_{360}-D_{540}**	Switched to other MC form	2 (1)	1.06 CI 95%:0.29-3.78	0 (0)	0 CI 95%:0-2.28	2 (1)	0.56 (CI 95%:0.31-0.08)	NA
**D_{360}-D_{540}**	Health Worsening	2 (1)	1 CI 95%:0.27-3.55	0 (0)	0 CI 95%:0-2.21	2 (1)	0.54 (CI 95%:0.26-0.08)	NA
**D_{360}-D_{540}**	Improved health	1 (1)	0.5 CI 95%:0.09-2.78	2 (1)	1.19 CI 95%:0.33-4.24	3 (1)	0.82 (CI 95%:0-0.1)	NA
**D_{360}-D_{540}**	Deceased	1 (0)	0.49 CI 95%:0.09-2.7	1 (1)	0.59 CI 95%:0.1-3.26	2 (1)	0.53 (CI 95%:0-0.08)	NA
**D_{360}-D_{540}**	Physician decision temporarily	1 (1)	0.52 CI 95%:0.09-2.91	0 (0)	0 CI 95%:0-2.28	1 (0)	0.28 (CI 95%:0.02-0.06)	NA
**D_{360}-D_{540}**	Other reason	1 (1)	0.52 CI 95%:0.09-2.88	0 (0)	0 CI 95%:0-2.28	1 (0)	0.28 (CI 95%:0.01-0.06)	NA
**D_{360}-D_{540}**	Physician decision permanently	1 (1)	0.52 CI 95%:0.09-2.89	0 (0)	0 CI 95%:0-2.28	1 (0)	0.28 (CI 95%:0.01-0.06)	NA
**D_{540}-D_{720}**	Health Worsening	2 (1)	1.07 CI 95%:0.29-3.82	1 (1)	0.61 CI 95%:0.11-3.39	3 (1)	0.86 (CI 95%:0.32-0.11)	NA
**D_{540}-D_{720}**	Physician decision permanently	1 (1)	0.54 CI 95%:0.1-3	3 (2)	1.9 CI 95%:0.65-5.43	4 (1)	1.17 (CI 95%:0.03-0.13)	NA
**D_{540}-D_{720}**	Switched to other non-MC treatment	1 (1)	0.54 CI 95%:0.1-3.01	1 (1)	0.64 CI 95%:0.11-3.52	2 (1)	0.59 (CI 95%:0.03-0.09)	NA

**D_{540}-D_{720}**	Ineffectiveness	1 (1)	0.54 CI 95%:0.09-2.98	0 (0)	0 CI 95%:0-2.32	1 (0)	0.29 (CI 95%:0.03-0.06)	NA
**D_{540}-D_{720}**	Improved health	0 (0)	0 CI 95%:0-2.01	1 (1)	0.62 CI 95%:0.11-3.41	1 (0)	0.29 (CI 95%:0-0.06)	NA
**D_{540}-D_{720}**	License expired	0 (0)	0 CI 95%:0-2.02	1 (1)	0.62 CI 95%:0.11-3.43	1 (0)	0.29 (CI 95%:0-0.06)	NA
**D_{540}-D_{720}**	Deceased	0 (0)	0 CI 95%:0-1.99	1 (1)	0.61 CI 95%:0.11-3.37	1 (0)	0.28 (CI 95%:0-0.06)	NA
**D_{720}-D_{900}**	License expired	1 (1)	0.56 CI 95%:0.1-3.08	1 (1)	0.65 CI 95%:0.11-3.56	2 (1)	0.6 (CI 95%:0.04-0.09)	NA
**D_{720}-D_{900}**	Improved health	1 (1)	0.55 CI 95%:0.1-3.05	0 (0)	0 CI 95%:0-2.4	1 (0)	0.3 (CI 95%:0.04-0.06)	NA
**D_{720}-D_{900}**	Intermittently continued	1 (1)	0.55 CI 95%:0.1-3.06	0 (0)	0 CI 95%:0-2.4	1 (0)	0.3 (CI 95%:0.04-0.06)	NA
**D_{720}-D_{900}**	Financial	1 (1)	0.55 CI 95%:0.1-3.03	0 (0)	0 CI 95%:0-2.39	1 (0)	0.29 (CI 95%:0.03-0.06)	NA
**D_{720}-D_{900}**	Physician decision permanently	0 (0)	0 CI 95%:0-2.09	1 (1)	0.65 CI 95%:0.11-3.59	1 (0)	0.3 (CI 95%:0-0.06)	NA
**D_{720}-D_{900}**	Health Worsening	0 (0)	0 CI 95%:0-2.06	1 (1)	0.64 CI 95%:0.11-3.54	1 (0)	0.29 (CI 95%:0-0.06)	NA
**D_{900}-D_{1080}**	Improved health	2 (1)	1.12 CI 95%:0.31-4	0 (0)	0 CI 95%:0-2.48	2 (1)	0.61 (CI 95%:0.38-0.09)	NA
**D_{900}-D_{1080}**	Health Worsening	0 (0)	0 CI 95%:0-2.09	2 (1)	1.32 CI 95%:0.36-4.67	2 (1)	0.6 (CI 95%:0-0.09)	NA
**D_{900}-D_{1080}**	Switched to other non-MC treatment	1 (1)	0.56 CI 95%:0.1-3.13	0 (0)	0 CI 95%:0-2.48	1 (0)	0.3 (CI 95%:0.05-0.06)	NA
**D_{900}-D_{1080}**	Immigration	0 (0)	0 CI 95%:0-2.09	1 (1)	0.66 CI 95%:0.12-3.66	1 (0)	0.3 (CI 95%:0-0.06)	NA
**D_{1080}-D_{1260}**	Improved health	2 (1)	1.14 CI 95%:0.31-4.07	3 (2)	2.03 CI 95%:0.69-5.79	5 (2)	1.55 (CI 95%:0.4-0.15)	NA
**D_{1080}-D_{1260}**	Other reason	1 (1)	0.57 CI 95%:0.1-3.18	0 (0)	0 CI 95%:0-2.55	1 (0)	0.31 (CI 95%:0.06-0.06)	NA

**D_{1080}- D_{1260}**	License expired	0 (0)	0 CI 95%:0-2.15	1 (1)	0.68 CI 95%:0.12-3.75	1 (0)	0.31 (CI 95%:0-0.06)	NA

MC, Medical cannabis; Numbers does not add to 100% due to concomitant reasons; Only time points with dropout reports are indicated.

Table S4: Specific sample etiologies/diagnoses for treatment

**Pain diagnosis etiology**	**Women n=607 N (%)**	**Women proportion (CI 95%)**	**Men n=513 N (%)**	**Men proportional (CI 95%)**	**Overall sample N=1120 N (%)**	**Overall proportion (CI 95%)**	**Wilson's X^2 test (p-value)**
**Chronic pain**	512 (84)	84.35 (CI 95%: 81.24-87.02)	407 (79)	79.34 (CI 95%: 75.62-82.62)	919 (82)	82.05 (CI 95%: 79.81-84.3)	4.41 (0.04)
**Neuropathic pain**	345 (57)	56.84 (CI 95%: 52.87-60.72)	353 (69)	68.81 (CI 95%: 64.67-72.67)	698 (62)	62.32 (CI 95%: 59.48-65.16)	16.47 (<0.001)
Lumbar Herniated Disc	187 (31)	30.81 (CI 95%: 27.26-34.59)	160 (31)	31.19 (CI 95%: 27.33-35.33)	347 (31)	30.98 (CI 95%: 28.27-33.69)	0.01 (0.94)
Cervical radicular pain	40 (7)	6.59 (CI 95%: 4.88-8.85)	45 (9)	8.77 (CI 95%: 6.62-11.54)	85 (8)	7.59 (CI 95%: 6.04-9.14)	1.59 (0.21)
Complex regional pain syndrome	31 (5)	5.11 (CI 95%: 3.62-7.16)	24 (5)	4.68 (CI 95%: 3.16-6.87)	55 (5)	4.91 (CI 95%: 3.65-6.18)	0.04 (0.85)
Diabetic Neuropathy Pain	25 (4)	4.12 (CI 95%: 2.81-6.01)	34 (7)	6.63 (CI 95%: 4.78-9.12)	59 (5)	5.27 (CI 95%: 3.96-6.58)	3.02 (0.08)
Nerve injury	21 (3)	3.46 (CI 95%: 2.27-5.23)	43 (8)	8.38 (CI 95%: 6.28-11.1)	64 (6)	5.71 (CI 95%: 4.35-7.07)	11.6 (<0.001)
MS related neuropathic pain	12 (2)	1.98 (CI 95%: 1.13-3.42)	5 (1)	0.97 (CI 95%: 0.42-2.26)	17 (2)	1.52 (CI 95%: 0.8-2.23)	1.26 (0.26)

Post herpetic neuralgia	9 (1)	1.48 (CI 95%: 0.78-2.79)	10 (2)	1.95 (CI 95%: 1.06-3.55)	19 (2)	1.7 (CI 95%: 0.94-2.45)	0.14 (0.71)
Chemotherapy induced neuropathic pain	10 (2)	1.65 (CI 95%: 0.9-3.01)	5 (1)	0.97 (CI 95%: 0.42-2.26)	15 (1)	1.34 (CI 95%: 0.67-2.01)	0.51 (0.48)
Trigeminal neuralgia	9 (1)	1.48 (CI 95%: 0.78-2.79)	1 (0)	0.19 (CI 95%: 0.03-1.1)	10 (1)	0.89 (CI 95%: 0.34-1.44)	NA
Idiopathic peripheral neuropathy	8 (1)	1.32 (CI 95%: 0.67-2.58)	9 (2)	1.75 (CI 95%: 0.93-3.3)	17 (2)	1.52 (CI 95%: 0.8-2.23)	0.12 (0.73)
Spinal cord injury	8 (1)	1.32 (CI 95%: 0.67-2.58)	8 (2)	1.56 (CI 95%: 0.79-3.05)	16 (1)	1.43 (CI 95%: 0.73-2.12)	0.01 (0.93)
Phantom limb pain	4 (1)	0.66 (CI 95%: 0.26-1.68)	20 (4)	3.9 (CI 95%: 2.54-5.94)	24 (2)	2.14 (CI 95%: 1.29-2.99)	NA
Small fiber neuropathy	3 (0)	0.49 (CI 95%: 0.17-1.44)	6 (1)	1.17 (CI 95%: 0.54-2.53)	9 (1)	0.8 (CI 95%: 0.28-1.33)	NA
Facial nerve injury	3 (0)	0.49 (CI 95%: 0.17-1.44)	4 (1)	0.78 (CI 95%: 0.3-1.99)	7 (1)	0.62 (CI 95%: 0.16-1.09)	NA
CVA related pain	3 (0)	0.49 (CI 95%: 0.17-1.44)	3 (1)	0.58 (CI 95%: 0.2-1.71)	6 (1)	0.54 (CI 95%: 0.11-0.96)	NA
Carpal tunnel syndrome	4 (1)	0.66 (CI 95%: 0.26-1.68)	1 (0)	0.19 (CI 95%: 0.03-1.1)	5 (0)	0.45 (CI 95%: 0.06-0.84)	NA
Parkinson's disease related peripheral neuropathy	3 (0)	0.49 (CI 95%: 0.17-1.44)	1 (0)	0.19 (CI 95%: 0.03-1.1)	4 (0)	0.36 (CI 95%: 0.01-0.71)	NA

Post mastectomy pain	4 (1)	0.66 (CI 95%: 0.26-1.68)	0 (0)	0 (CI 95%: 0-0.74)	4 (0)	0.36 (CI 95%: 0.01-0.71)	NA
Failed back surgery pain	2 (0)	0.33 (CI 95%: 0.09-1.19)	4 (1)	0.78 (CI 95%: 0.3-1.99)	6 (1)	0.54 (CI 95%: 0.11-0.96)	NA
Cancer related neuropathic pain	2 (0)	0.33 (CI 95%: 0.09-1.19)	0 (0)	0 (CI 95%: 0-0.74)	2 (0)	0.18 (CI 95%: -0.07-0.43)	NA
Post lumpectomy pain	2 (0)	0.33 (CI 95%: 0.09-1.19)	0 (0)	0 (CI 95%: 0-0.74)	2 (0)	0.18 (CI 95%: -0.07-0.43)	NA
Berger's disease	1 (0)	0.16 (CI 95%: 0.03-0.93)	2 (0)	0.39 (CI 95%: 0.11-1.41)	3 (0)	0.27 (CI 95%: -0.03-0.57)	NA
Radiation therapy related neuropathic pain	1 (0)	0.16 (CI 95%: 0.03-0.93)	2 (0)	0.39 (CI 95%: 0.11-1.41)	3 (0)	0.27 (CI 95%: -0.03-0.57)	NA
Transverse Myelitis	1 (0)	0.16 (CI 95%: 0.03-0.93)	2 (0)	0.39 (CI 95%: 0.11-1.41)	3 (0)	0.27 (CI 95%: -0.03-0.57)	NA
**Musculoskeletal pain**	197 (32)	32.45 (CI 95%: 28.85-36.28)	139 (27)	27.1 (CI 95%: 23.43-31.1)	336 (30)	30 (CI 95%: 27.32-32.68)	3.55 (0.06)
Osteoarthritis	45 (7)	7.41 (CI 95%: 5.59-9.78)	14 (3)	2.73 (CI 95%: 1.63-4.53)	59 (5)	5.27 (CI 95%: 3.96-6.58)	11.3 (0)
Rheumatoid Arthritis	38 (6)	6.26 (CI 95%: 4.59-8.48)	10 (2)	1.95 (CI 95%: 1.06-3.55)	48 (4)	4.29 (CI 95%: 3.1-5.47)	11.6 (0)
Spinal stenosis	22 (4)	3.62 (CI 95%: 2.41-5.43)	19 (4)	3.7 (CI 95%: 2.38-5.71)	41 (4)	3.66 (CI 95%: 2.56-4.76)	0 (1)

Low back pain	17 (3)	2.8 (CI 95%: 1.76-4.44)	11 (2)	2.14 (CI 95%: 1.2-3.8)	28 (3)	2.5 (CI 95%: 1.59-3.41)	0.26 (0.61)
Tendon tear	14 (2)	2.31 (CI 95%: 1.38-3.83)	14 (3)	2.73 (CI 95%: 1.63-4.53)	28 (3)	2.5 (CI 95%: 1.59-3.41)	0.07 (0.8)
Unknown origin shoulder pain	9 (1)	1.48 (CI 95%: 0.78-2.79)	10 (2)	1.95 (CI 95%: 1.06-3.55)	19 (2)	1.7 (CI 95%: 0.94-2.45)	0.14 (0.71)
Unknown origin knee pain	8 (1)	1.32 (CI 95%: 0.67-2.58)	8 (2)	1.56 (CI 95%: 0.79-3.05)	16 (1)	1.43 (CI 95%: 0.73-2.12)	0.01 (0.93)
Bones cancer pain	8 (1)	1.32 (CI 95%: 0.67-2.58)	5 (1)	0.97 (CI 95%: 0.42-2.26)	13 (1)	1.16 (CI 95%: 0.53-1.79)	0.06 (0.8)
Psoriatic arthritis	8 (1)	1.32 (CI 95%: 0.67-2.58)	4 (1)	0.78 (CI 95%: 0.3-1.99)	12 (1)	1.07 (CI 95%: 0.47-1.67)	NA
Scoliosis related pain	6 (1)	0.99 (CI 95%: 0.45-2.14)	3 (1)	0.58 (CI 95%: 0.2-1.71)	9 (1)	0.8 (CI 95%: 0.28-1.33)	NA
Ankylosing spondylitis	5 (1)	0.82 (CI 95%: 0.35-1.91)	6 (1)	1.17 (CI 95%: 0.54-2.53)	11 (1)	0.98 (CI 95%: 0.4-1.56)	0.08 (0.78)
Multi trauma related pain	5 (1)	0.82 (CI 95%: 0.35-1.91)	4 (1)	0.78 (CI 95%: 0.3-1.99)	9 (1)	0.8 (CI 95%: 0.28-1.33)	NA
Hip replacement pain	5 (1)	0.82 (CI 95%: 0.35-1.91)	1 (0)	0.19 (CI 95%: 0.03-1.1)	6 (1)	0.54 (CI 95%: 0.11-0.96)	NA
Unknown origin hip pain	3 (0)	0.49 (CI 95%: 0.17-1.44)	5 (1)	0.97 (CI 95%: 0.42-2.26)	8 (1)	0.71 (CI 95%: 0.22-1.21)	NA

Post traumatic pain	4 (1)	0.66 (CI 95%: 0.26-1.68)	4 (1)	0.78 (CI 95%: 0.3-1.99)	8 (1)	0.71 (CI 95%: 0.22-1.21)	NA
Unknown origin neck pain	4 (1)	0.66 (CI 95%: 0.26-1.68)	4 (1)	0.78 (CI 95%: 0.3-1.99)	8 (1)	0.71 (CI 95%: 0.22-1.21)	NA
Knee replacement pain	3 (0)	0.49 (CI 95%: 0.17-1.44)	3 (1)	0.58 (CI 95%: 0.2-1.71)	6 (1)	0.54 (CI 95%: 0.11-0.96)	NA
Pathologic fracture	3 (0)	0.49 (CI 95%: 0.17-1.44)	2 (0)	0.39 (CI 95%: 0.11-1.41)	5 (0)	0.45 (CI 95%: 0.06-0.84)	NA
Kyphosis related pain	3 (0)	0.49 (CI 95%: 0.17-1.44)	1 (0)	0.19 (CI 95%: 0.03-1.1)	4 (0)	0.36 (CI 95%: 0.01-0.71)	NA
Unknown origin Pelvic pain	3 (0)	0.49 (CI 95%: 0.17-1.44)	0 (0)	0 (CI 95%: 0-0.74)	3 (0)	0.27 (CI 95%: -0.03-0.57)	NA
Osteomyelitis	2 (0)	0.33 (CI 95%: 0.09-1.19)	0 (0)	0 (CI 95%: 0-0.74)	2 (0)	0.18 (CI 95%: -0.07-0.43)	NA
Unknown origin joint pain	1 (0)	0.16 (CI 95%: 0.03-0.93)	7 (1)	1.36 (CI 95%: 0.66-2.79)	8 (1)	0.71 (CI 95%: 0.22-1.21)	NA
Parkinson related pain	1 (0)	0.16 (CI 95%: 0.03-0.93)	2 (0)	0.39 (CI 95%: 0.11-1.41)	3 (0)	0.27 (CI 95%: -0.03-0.57)	NA
Unknown origin legs pain	1 (0)	0.16 (CI 95%: 0.03-0.93)	2 (0)	0.39 (CI 95%: 0.11-1.41)	3 (0)	0.27 (CI 95%: -0.03-0.57)	NA
FMF arthritis	1 (0)	0.16 (CI 95%: 0.03-0.93)	0 (0)	0 (CI 95%: 0-0.74)	1 (0)	0.09 (CI 95%: -0.09-0.26)	NA

Gout	0 (0)	0 (CI 95%: 0-0.63)	5 (1)	0.97 (CI 95%: 0.42-2.26)	5 (0)	0.45 (CI 95%: 0.06-0.84)	NA
**Nociplastic pain**	131 (22)	21.58 (CI 95%: 18.49-25.03)	29 (6)	5.65 (CI 95%: 3.96-8)	160 (14)	14.29 (CI 95%: 12.24-16.34)	56.315 (<0.001)
Fibromyalgia	130 (21)	21.42 (CI 95%: 18.34-24.85)	28 (5)	5.46 (CI 95%: 3.8-7.78)	158 (14)	14.11 (CI 95%: 12.07-16.15)	57.1 (<0.001)
**Visceral pain**	76 (13)	12.52 (CI 95%: 10.12-15.39)	8 (2)	1.56 (CI 95%: 0.79-3.05)	84 (8)	7.5 (CI 95%: 5.96-9.04)	46.583 (<0.001)
Endometriosis	56 (9)	9.23 (CI 95%: 7.17-11.79)	NA	NA	56 (5)	5 (CI 95%: 3.72-6.28)	NA
Adenomyosis	5 (1)	0.82 (CI 95%: 0.35-1.91)	0 (0)	0 (CI 95%: 0-0.74)	5 (0)	0.45 (CI 95%: 0.06-0.84)	NA
Interstitial cystitis	3 (0)	0.49 (CI 95%: 0.17-1.44)	0 (0)	0 (CI 95%: 0-0.74)	3 (0)	0.27 (CI 95%: 0-0.57)	NA
Post abdominal surgery pain	2 (0)	0.33 (CI 95%: 0.09-1.19)	1 (0)	0.19 (CI 95%: 0.03-1.1)	3 (0)	0.27 (CI 95%: 0-0.57)	NA
Ascites related pain	2 (0)	0.33 (CI 95%: 0.09-1.19)	0 (0)	0 (CI 95%: 0-0.74)	2 (0)	0.18 (CI 95%: 0-0.43)	NA
Perianal pain	1 (0)	0.16 (CI 95%: 0.03-0.93)	2 (0)	0.39 (CI 95%: 0.11-1.41)	3 (0)	0.27 (CI 95%: 0-0.57)	NA
IBD related abdominal pain	1 (0)	0.16 (CI 95%: 0.03-0.93)	1 (0)	0.19 (CI 95%: 0.03-1.1)	2 (0)	0.18 (CI 95%: 0-0.43)	NA

Achalasia	1 (0)	0.16 (CI 95%: 0.03-0.93)	0 (0)	0 (CI 95%: 0-0.74)	1 (0)	0.09 (CI 95%: 0-0.26)	NA
Anal pain	1 (0)	0.16 (CI 95%: 0.03-0.93)	0 (0)	0 (CI 95%: 0-0.74)	1 (0)	0.09 (CI 95%: 0-0.26)	NA
Cancer related abdominal pain	1 (0)	0.16 (CI 95%: 0.03-0.93)	0 (0)	0 (CI 95%: 0-0.74)	1 (0)	0.09 (CI 95%: 0-0.26)	NA
**Headaches**	45 (7)	7.41 (CI 95%: 5.59-9.78)	22 (4)	4.29 (CI 95%: 2.85-6.41)	67 (6)	5.98 (CI 95%: 4.59-7.37)	4.29 (0.04)
Migraine	22 (4)	3.62 (CI 95%: 2.41-5.43)	3 (1)	0.58 (CI 95%: 0.2-1.71)	25 (2)	2.23 (CI 95%: 1.37-3.1)	10.4 (<0.001)
Other	7 (1)	1.15 (CI 95%: 0.56-2.36)	3 (1)	0.58 (CI 95%: 0.2-1.71)	10 (1)	0.89 (CI 95%: 0.34-1.44)	0.47 (0.49)
Post-surgical headache	3 (0)	0.49 (CI 95%: 0.17-1.44)	3 (1)	0.58 (CI 95%: 0.2-1.71)	6 (1)	0.54 (CI 95%: 0.11-0.96)	NA
Hemangioma related headache	3 (0)	0.49 (CI 95%: 0.17-1.44)	1 (0)	0.19 (CI 95%: 0.03-1.1)	4 (0)	0.36 (CI 95%: 0.01-0.71)	NA
Cervicogenic headache	2 (0)	0.33 (CI 95%: 0.09-1.19)	3 (1)	0.58 (CI 95%: 0.2-1.71)	5 (0)	0.45 (CI 95%: 0.06-0.84)	NA
Post-concussion headache	1 (0)	0.16 (CI 95%: 0.03-0.93)	4 (1)	0.78 (CI 95%: 0.3-1.99)	5 (0)	0.45 (CI 95%: 0.06-0.84)	NA
Tension headache	1 (0)	0.16 (CI 95%: 0.03-0.93)	3 (1)	0.58 (CI 95%: 0.2-1.71)	4 (0)	0.36 (CI 95%: 0.01-0.71)	NA

Trigeminal neuralgia	1 (0)	0.16 (CI 95%: 0.03-0.93)	1 (0)	0.19 (CI 95%: 0.03-1.1)	2 (0)	0.18 (CI 95%: 0-0.43)	NA
Cluster headache	1 (0)	0.16 (CI 95%: 0.03-0.93)	0 (0)	0 (CI 95%: 0-0.74)	1 (0)	0.09 (CI 95%: 0-0.26)	NA
**Oncology**	43 (7)	7.08 (CI 95%: 5.3-9.41)	47 (9)	9.16 (CI 95%: 6.96-11.97)	90 (8)	8.04 (CI 95%: 6.44-9.63)	1.36 (0.24)
Breast	10 (2)	1.65 (CI 95%: 0.9-3.01)	1 (0)	0.19 (CI 95%: 0.03-1.1)	11 (1)	0.98 (CI 95%: 0.4-1.56)	NA
Other	8 (1)	1.32 (CI 95%: 0.67-2.58)	4 (1)	0.78 (CI 95%: 0.3-1.99)	12 (1)	1.07 (CI 95%: 0.47-1.67)	NA
Pancreas	3 (0)	0.49 (CI 95%: 0.17-1.44)	8 (2)	1.56 (CI 95%: 0.79-3.05)	11 (1)	0.98 (CI 95%: 0.4-1.56)	NA
Colon	3 (0)	0.49 (CI 95%: 0.17-1.44)	5 (1)	0.97 (CI 95%: 0.42-2.26)	8 (1)	0.71 (CI 95%: 0.22-1.21)	NA
Multiple myeloma	3 (0)	0.49 (CI 95%: 0.17-1.44)	4 (1)	0.78 (CI 95%: 0.3-1.99)	7 (1)	0.62 (CI 95%: 0.16-1.09)	NA
Lungs	4 (1)	0.66 (CI 95%: 0.26-1.68)	3 (1)	0.58 (CI 95%: 0.2-1.71)	7 (1)	0.62 (CI 95%: 0.16-1.09)	NA
Endometrium	4 (1)	0.66 (CI 95%: 0.26-1.68)	NA	NA	4 (0)	0.36 (CI 95%: 0.01-0.71)	NA
Leukemia	2 (0)	0.33 (CI 95%: 0.09-1.19)	2 (0)	0.39 (CI 95%: 0.11-1.41)	4 (0)	0.36 (CI 95%: 0.01-0.71)	NA

Lymphoma	2 (0)	0.33 (CI 95%: 0.09-1.19)	2 (0)	0.39 (CI 95%: 0.11-1.41)	4 (0)	0.36 (CI 95%: 0.01-0.71)	NA
Bladder	1 (0)	0.16 (CI 95%: 0.03-0.93)	2 (0)	0.39 (CI 95%: 0.11-1.41)	3 (0)	0.27 (CI 95%: 0-0.57)	NA
Kidney	1 (0)	0.16 (CI 95%: 0.03-0.93)	2 (0)	0.39 (CI 95%: 0.11-1.41)	3 (0)	0.27 (CI 95%: 0-0.57)	NA
Skin	1 (0)	0.16 (CI 95%: 0.03-0.93)	0 (0)	0 (CI 95%: 0-0.74)	1 (0)	0.09 (CI 95%: 0-0.26)	NA
Stomach	1 (0)	0.16 (CI 95%: 0.03-0.93)	0 (0)	0 (CI 95%: 0-0.74)	1 (0)	0.09 (CI 95%: 0-0.26)	NA
Liver	0 (0)	0 (CI 95%: 0-0.63)	1 (0)	0.19 (CI 95%: 0.03-1.1)	1 (0)	0.09 (CI 95%: 0-0.26)	NA
Mouth and neck	0 (0)	0 (CI 95%: 0-0.63)	1 (0)	0.19 (CI 95%: 0.03-1.1)	1 (0)	0.09 (CI 95%: 0-0.26)	NA
Thyroid	0 (0)	0 (CI 95%: 0-0.63)	1 (0)	0.19 (CI 95%: 0.03-1.1)	1 (0)	0.09 (CI 95%: 0-0.26)	NA
Prostate	NA	NA	11 (2)	2.14 (CI 95%: 1.2-3.8)	11 (1)	0.98 (CI 95%: 0.4-1.56)	NA
**inflammatory bowel diseases**	27 (4)	4.45 (CI 95%: 3.07-6.39)	10 (2)	1.95 (CI 95%: 1.06-3.55)	37 (3)	3.3 (CI 95%: 2.26-4.35)	4.68 (0.03)
Crohn's Disease	16 (3)	2.64 (CI 95%: 1.63-4.24)	7 (1)	1.36 (CI 95%: 0.66-2.79)	23 (2)	2.05 (CI 95%: 1.22-2.88)	1.65 (0.2)

IBS	6 (1)	0.99 (CI 95%: 0.45-2.14)	1 (0)	0.19 (CI 95%: 0.03-1.1)	7 (1)	0.62 (CI 95%: 0.16-1.09)	NA
Ulcerative Colitis	5 (1)	0.82 (CI 95%: 0.35-1.91)	2 (0)	0.39 (CI 95%: 0.11-1.41)	7 (1)	0.62 (CI 95%: 0.16-1.09)	NA
Colon prolapse	1 (0)	0.16 (CI 95%: 0.03-0.93)	0 (0)	0 (CI 95%: 0-0.74)	1 (0)	0.09 (CI 95%: 0-0.26)	NA
Reflux	1 (0)	0.16 (CI 95%: 0.03-0.93)	0 (0)	0 (CI 95%: 0-0.74)	1 (0)	0.09 (CI 95%: 0-0.26)	NA
**Neurology**	39 (6)	6.43 (CI 95%: 4.74-8.66)	34 (7)	6.63 (CI 95%: 4.78-9.12)	73 (7)	6.52 (CI 95%: 5.07-7.96)	0 (0.99)
MS	26 (4)	4.28 (CI 95%: 2.94-6.2)	12 (2)	2.34 (CI 95%: 1.34-4.04)	38 (3)	3.39 (CI 95%: 2.33-4.45)	2.64 (0.1)
Parkinson disease	24 (4)	3.95 (CI 95%: 2.67-5.82)	15 (3)	2.92 (CI 95%: 1.78-4.77)	39 (3)	3.48 (CI 95%: 2.41-4.56)	0.6 (0.44)
Other	5 (1)	0.82 (CI 95%: 0.35-1.91)	11 (2)	2.14 (CI 95%: 1.2-3.8)	16 (1)	1.43 (CI 95%: 0.73-2.12)	2.57 (0.11)
Epilepsy	3 (0)	0.49 (CI 95%: 0.17-1.44)	2 (0)	0.39 (CI 95%: 0.11-1.41)	5 (0)	0.45 (CI 95%: 0.06-0.84)	NA
Essential tremor	4 (1)	0.66 (CI 95%: 0.26-1.68)	0 (0)	0 (CI 95%: 0-0.74)	4 (0)	0.36 (CI 95%: 0.01-0.71)	NA
Dementia	1 (0)	0.16 (CI 95%: 0.03-0.93)	2 (0)	0.39 (CI 95%: 0.11-1.41)	3 (0)	0.27 (CI 95%: 0-0.57)	NA

Neurofibromatosis	1 (0)	0.16 (CI 95%: 0.03-0.93)	2 (0)	0.39 (CI 95%: 0.11-1.41)	3 (0)	0.27 (CI 95%: 0-0.57)	NA
Tourette syndrome	1 (0)	0.16 (CI 95%: 0.03-0.93)	3 (1)	0.58 (CI 95%: 0.2-1.71)	4 (0)	0.36 (CI 95%: 0.01-0.71)	NA
Post CVA spasticity	1 (0)	0.16 (CI 95%: 0.03-0.93)	1 (0)	0.19 (CI 95%: 0.03-1.1)	2 (0)	0.18 (CI 95%: 0-0.43)	NA
ALS	1 (0)	0.16 (CI 95%: 0.03-0.93)	0 (0)	0 (CI 95%: 0-0.74)	1 (0)	0.09 (CI 95%: 0-0.26)	NA
**Post-traumatic stress disorder**	5 (1)	0.82 (CI 95%: 0.35-1.91)	31 (6)	6.04 (CI 95%: 4.29-8.45)	36 (3)	3.21 (CI 95%: 2.18-4.25)	22.7 (<0.001)
**Palliative conditions**	7 (1)	1.15 (CI 95%: 0.56-2.36)	8 (2)	1.56 (CI 95%: 0.79-3.05)	15 (1)	1.34 (CI 95%: 0.67-2.01)	0.11 (0.74)

ALS, amyotrophic lateral sclerosis; CVA, cerebrovascular accident; FMF, familial Mediterranean fever; IBD, inflammatory bowel disease; IBS, irritable bowel syndrome; MS, multiple sclerosis; NA, not applicable; Numbers does not add up to 100% due to concomitant diagnoses.

Table S5: Specific comorbidities diagnoses

Disease	Women n=607 N (%)	Women proportion (CI 95%)	Men n=513 N (%)	Men proportional (CI 95%)	Overall sample N=1120 N (%)	Overall proportion (CI 95%)	Wilson's X^2 test (p-value)
**Comorbidity diagnoses at baseline**							
Yes	435 (72)	71.66 (CI 95%:67.95 - 75.1)	358 (70)	69.79 (CI 95%:65.68 - 73.6)	793 (71)	70.8 (CI 95%:68.07 - 73.39)	0.39 (0.53)
No	159 (26)	26.19 (CI 95%:22.85 - 29.83)	147 (29)	28.65 (CI 95%:24.91 - 32.71)	306 (27)	27.32 (CI 95%:24.79 - 30.01)	0.73 (0.39)
**Specific diagnoses**							
HTN	232 (38)	38.22 (CI 95%: 34.44 - 42.15)	209 (41)	40.74 (CI 95%: 36.57 - 45.05)	441 (39)	39.38 (CI 95%: 36.55 - 42.27)	0.64 (0.42)
Diabetes	97 (16)	15.98 (CI 95%: 13.28 - 19.11)	128 (25)	24.95 (CI 95%: 21.4 - 28.87)	225 (20)	20.09 (CI 95%: 17.84 - 22.54)	13.4 (<0.001)
Psychiatric	91 (15)	14.99 (CI 95%: 12.37 - 18.05)	56 (11)	10.92 (CI 95%: 8.5 - 13.91)	147 (13)	13.13 (CI 95%: 11.27 - 15.23)	3.7 (0.05)
Hypothyroidism	73 (12)	12.03 (CI 95%: 9.67 - 14.86)	26 (5)	5.07 (CI 95%: 3.48 -7.32)	99 (9)	8.84 (CI 95%: 7.31 -10.65)	15.9 (<0.001)
Hyperlipidemia	72 (12)	11.86 (CI 95%: 9.53 - 14.68)	95 (19)	18.52 (CI 95%: 15.4 - 22.11)	167 (15)	14.91 (CI 95%: 12.94 - 17.12)	9.19 (<0.005)

Respiratory	56 (9)	9.23 (CI 95%: 7.17 -11.79)	33 (6)	6.43 (CI 95%: 4.62 -8.9)	89 (8)	7.95 (CI 95%: 6.5 -9.68)	2.6 (0.11)
Depression	55 (9)	9.06 (CI 95%:7.03 -11.61)	28 (5)	5.46 (CI 95%:3.8 -7.78)	83 (7)	7.41 (CI 95%:6.01 -9.1)	4.75 (0.03)
Cardiac disease	50 (8)	8.24 (CI 95%: 6.3 -10.7)	71 (14)	13.84 (CI 95%: 11.12 -17.1)	121 (11)	10.8 (CI 95%: 9.11 -12.76)	8.49 (<0.005)
Osteoporosis	41 (7)	6.75 (CI 95%: 5.02 -9.04)	9 (2)	1.75 (CI 95%: 0.93 -3.3)	50 (4)	4.46 (CI 95%: 3.39 -5.85)	15.1 (<0.001)
Asthma	33 (5)	5.44(CI 95%:3.9 -7.54)	11 (2)	2.14(CI 95%:1.2 -3.8)	44 (4)	3.93(CI 95%:2.93 -5.24)	7.14(<0.01)
Anxiety	27 (4)	4.45 (CI 95%:3.07 -6.39)	13 (3)	2.53 (CI 95%:1.49 -4.29)	40 (4)	3.57 (CI 95%:2.62 -4.84)	2.43 (0.12)
IHD	11 (2)	1.81(CI 95%:1.01 -3.22)	28 (5)	5.46(CI 95%:3.8 -7.78)	39 (3)	3.48(CI 95%:2.55 -4.74)	9.94(<0.005)
AF	9 (1)	1.48(CI 95%:0.78 -2.79)	8 (2)	1.56(CI 95%:0.79 -3.05)	17 (2)	1.52(CI 95%:0.93 -2.44)	0(1)
CRF	8 (1)	1.32 (CI 95%: 0.67 -2.58)	9 (2)	1.75 (CI 95%: 0.93 -3.3)	17 (2)	1.52 (CI 95%: 0.93 -2.44)	0.12 (0.73)
ADHD	7 (1)	1.15 (CI 95%: 0.56 -2.36)	8 (2)	1.56 (CI 95%: 0.79 -3.05)	15 (1)	1.34 (CI 95%: 0.79 -2.22)	0.11 (0.74)
COPD	7 (1)	1.15(CI 95%:0.56 -2.36)	6 (1)	1.17(CI 95%:0.54 -2.53)	13 (1)	1.16(CI 95%:0.66 -2)	0(1)

S/P CVA	6 (1)	0.99 (CI 95%: 0.45 -2.14)	6 (1)	1.17 (CI 95%: 0.54 -2.53)	12 (1)	1.07 (CI 95%: 0.59 -1.89)	0 (1)
Anemia	6 (1)	0.99 (CI 95%: 0.45 -2.14)	3 (1)	0.58 (CI 95%: 0.2 -1.71)	9 (1)	0.8 (CI 95%: 0.4 -1.55)	NA
Sarcoidosis	6 (1)	0.99(CI 95%:0.45 - 2.14)	2 (0)	0.39(CI 95%:0.11 - 1.41)	8 (1)	0.71(CI 95%:0.34 - 1.43)	NA
Glaucoma	5 (1)	0.82 (CI 95%: 0.35 -1.91)	4 (1)	0.78 (CI 95%: 0.3 -1.99)	9 (1)	0.8 (CI 95%: 0.4 -1.55)	NA
Urine incontinences	5 (1)	0.82 (CI 95%: 0.35 -1.91)	0 (0)	0 (CI 95%: 0 - 0.74)	5 (0)	0.45 (CI 95%: 0.16 -1.07)	NA
Arrhythmia	4 (1)	0.66(CI 95%:0.26 - 1.68)	6 (1)	1.17(CI 95%:0.54 - 2.53)	10 (1)	0.89(CI 95%:0.46 - 1.66)	NA
Thrombophilia	3 (0)	0.49 (CI 95%: 0.17 -1.44)	4 (1)	0.78 (CI 95%: 0.3 -1.99)	7 (1)	0.63 (CI 95%: 0.27 -1.31)	NA
Cardiac pacemaker	3 (0)	0.49(CI 95%:0.17 - 1.44)	2 (0)	0.39(CI 95%:0.11 - 1.41)	5 (0)	0.45(CI 95%:0.16 - 1.07)	NA
Hashimoto's thyroiditis	4 (1)	0.66 (CI 95%: 0.26 -1.68)	1 (0)	0.19 (CI 95%: 0.03 -1.1)	5 (0)	0.45 (CI 95%: 0.16 -1.07)	NA
Reflux	4 (1)	0.66 (CI 95%: 0.26 -1.68)	1 (0)	0.19 (CI 95%: 0.03 -1.1)	5 (0)	0.45 (CI 95%: 0.16 -1.07)	NA
Overactive bladder	3 (0)	0.49 (CI 95%: 0.17 -1.44)	1 (0)	0.19 (CI 95%: 0.03 -1.1)	4 (0)	0.36 (CI 95%: 0.1 -0.95)	NA
DVT	4 (1)	0.66 (CI 95%: 0.26 -1.68)	1 (0)	0.19 (CI 95%: 0.03 -1.1)	5 (0)	0.45 (CI 95%: 0.16 -1.07)	NA
Cataract	3 (0)	0.49 (CI 95%: 0.17 -1.44)	1 (0)	0.19 (CI 95%: 0.03 -1.1)	4 (0)	0.36 (CI 95%: 0.1 -0.95)	NA

Diaphragmatic hernia	3 (0)	0.49 (CI 95%: 0.17 -1.44)	0 (0)	0 (CI 95%: 0 - 0.74)	3 (0)	0.27 (CI 95%: 0.05 -0.82)	NA
S/P breast cancer	3 (0)	0.49 (CI 95%: 0.17 -1.44)	0 (0)	0 (CI 95%: 0 - 0.74)	3 (0)	0.27 (CI 95%: 0.05 -0.82)	NA
Spinal stenosis	3 (0)	0.49 (CI 95%: 0.17 -1.44)	0 (0)	0 (CI 95%: 0 - 0.74)	3 (0)	0.27 (CI 95%: 0.05 -0.82)	NA
Scoliosis	3 (0)	0.49 (CI 95%: 0.17 -1.44)	0 (0)	0 (CI 95%: 0 - 0.74)	3 (0)	0.27 (CI 95%: 0.05 -0.82)	NA
Mitral valve insufficiency	3 (0)	0.49(CI 95%:0.17 - 1.44)	0 (0)	0(CI 95%:0 - 0.74)	3 (0)	0.27(CI 95%:0.05 - 0.82)	NA
Aortic stenosis	3 (0)	0.49(CI 95%:0.17 - 1.44)	0 (0)	0(CI 95%:0 - 0.74)	3 (0)	0.27(CI 95%:0.05 - 0.82)	NA
CHF	2 (0)	0.33(CI 95%:0.09 - 1.19)	5 (1)	0.97(CI 95%:0.42 - 2.26)	7 (1)	0.63(CI 95%:0.27 - 1.31)	NA
PVD	2 (0)	0.33 (CI 95%: 0.09 -1.19)	2 (0)	0.39 (CI 95%: 0.11 -1.41)	4 (0)	0.36 (CI 95%: 0.1 -0.95)	NA
Gastritis	2 (0)	0.33 (CI 95%: 0.09 -1.19)	3 (1)	0.58 (CI 95%: 0.2 -1.71)	5 (0)	0.45 (CI 95%: 0.16 -1.07)	NA
Sarcoidosis	2 (0)	0.33 (CI 95%: 0.09 -1.19)	2 (0)	0.39 (CI 95%: 0.11 -1.41)	4 (0)	0.36 (CI 95%: 0.1 -0.95)	NA
Pulmonary hypertension	2 (0)	0.33(CI 95%:0.09 - 1.19)	2 (0)	0.39(CI 95%:0.11 - 1.41)	4 (0)	0.36(CI 95%:0.1 - 0.95)	NA
Hypokalemia	2 (0)	0.33 (CI 95%: 0.09 -1.19)	1 (0)	0.19 (CI 95%: 0.03 -1.1)	3 (0)	0.27 (CI 95%: 0.05 -0.82)	NA

FMF	2 (0)	0.33 (CI 95%: 0.09 -1.19)	1 (0)	0.19 (CI 95%: 0.03 -1.1)	3 (0)	0.27 (CI 95%: 0.05 -0.82)	NA
Psoriasis	2 (0)	0.33 (CI 95%: 0.09 -1.19)	1 (0)	0.19 (CI 95%: 0.03 -1.1)	3 (0)	0.27 (CI 95%: 0.05 -0.82)	NA
HCV	2 (0)	0.33 (CI 95%: 0.09 -1.19)	1 (0)	0.19 (CI 95%: 0.03 -1.1)	3 (0)	0.27 (CI 95%: 0.05 -0.82)	NA
Aortic valve replacement	2 (0)	0.33(CI 95%:0.09 -1.19)	1 (0)	0.19(CI 95%:0.03 -1.1)	3 (0)	0.27(CI 95%:0.05 -0.82)	NA
PE	2 (0)	0.33(CI 95%:0.09 -1.19)	1 (0)	0.19(CI 95%:0.03 -1.1)	3 (0)	0.27(CI 95%:0.05 -0.82)	NA
Bipolar disorder	2 (0)	0.33 (CI 95%:0.09 -1.19)	1 (0)	0.19 (CI 95%:0.03 -1.1)	3 (0)	0.27 (CI 95%:0.05 -0.82)	NA
Amyloidosis	2 (0)	0.33 (CI 95%: 0.09 -1.19)	0 (0)	0 (CI 95%: 0 -0.74)	2 (0)	0.18 (CI 95%: 0 -0.69)	NA
Anal fissure	2 (0)	0.33 (CI 95%: 0.09 -1.19)	0 (0)	0 (CI 95%: 0 -0.74)	2 (0)	0.18 (CI 95%: 0 -0.69)	NA
Fatty liver	2 (0)	0.33 (CI 95%: 0.09 -1.19)	0 (0)	0 (CI 95%: 0 -0.74)	2 (0)	0.18 (CI 95%: 0 -0.69)	NA
Retinal detachment	2 (0)	0.33 (CI 95%: 0.09 -1.19)	0 (0)	0 (CI 95%: 0 -0.74)	2 (0)	0.18 (CI 95%: 0 -0.69)	NA
Esophageal reflux	2 (0)	0.33 (CI 95%: 0.09 -1.19)	0 (0)	0 (CI 95%: 0 -0.74)	2 (0)	0.18 (CI 95%: 0 -0.69)	NA
Vertigo	2 (0)	0.33 (CI 95%: 0.09 -1.19)	0 (0)	0 (CI 95%: 0 -0.74)	2 (0)	0.18 (CI 95%: 0 -0.69)	NA
S/P breast cancer-lumpectomy	2 (0)	0.33 (CI 95%: 0.09 -1.19)	0 (0)	0 (CI 95%: 0 -0.74)	2 (0)	0.18 (CI 95%: 0 -0.69)	NA

Thrombocytopenia	2 (0)	0.33 (CI 95%: 0.09 -1.19)	0 (0)	0 (CI 95%: 0 - 0.74)	2 (0)	0.18 (CI 95%: 0 -0.69)	NA
Celiac	2 (0)	0.33 (CI 95%: 0.09 -1.19)	0 (0)	0 (CI 95%: 0 - 0.74)	2 (0)	0.18 (CI 95%: 0 -0.69)	NA
Menopause symptoms	2 (0)	0.33 (CI 95%: 0.09 -1.19)	0 (0)	0 (CI 95%: 0 - 0.74)	2 (0)	0.18 (CI 95%: 0 -0.69)	NA
Ehlers-Danlos syndrome	2 (0)	0.33 (CI 95%: 0.09 -1.19)	0 (0)	0 (CI 95%: 0 - 0.74)	2 (0)	0.18 (CI 95%: 0 -0.69)	NA
S/P SVT Post ablation	2 (0)	0.33(CI 95%:0.09 - 1.19)	0 (0)	0(CI 95%:0 - 0.74)	2 (0)	0.18(CI 95%:0 -0.69)	NA
Chronic bronchitis	2 (0)	0.33(CI 95%:0.09 - 1.19)	0 (0)	0(CI 95%:0 - 0.74)	2 (0)	0.18(CI 95%:0 -0.69)	NA
S/P MI	1 (0)	0.16(CI 95%:0.03 - 0.93)	7 (1)	1.36(CI 95%:0.66 - 2.79)	8 (1)	0.71(CI 95%:0.34 - 1.43)	NA
S/P PTCA	1 (0)	0.16(CI 95%:0.03 - 0.93)	5 (1)	0.97(CI 95%:0.42 - 2.26)	6 (1)	0.54(CI 95%:0.22 - 1.19)	NA
OSA	2 (0)	0.33(CI 95%:0.09 - 1.19)	6 (1)	1.17(CI 95%:0.54 - 2.53)	8 (1)	0.71(CI 95%:0.34 - 1.43)	NA
Dry eyes	1 (0)	0.16 (CI 95%: 0.03 -0.93)	2 (0)	0.39 (CI 95%: 0.11 -1.41)	3 (0)	0.27 (CI 95%: 0.05 -0.82)	NA
Dialysis	1 (0)	0.16 (CI 95%: 0.03 -0.93)	2 (0)	0.39 (CI 95%: 0.11 -1.41)	3 (0)	0.27 (CI 95%: 0.05 -0.82)	NA
Renal transplant	1 (0)	0.16 (CI 95%: 0.03 -0.93)	2 (0)	0.39 (CI 95%: 0.11 -1.41)	3 (0)	0.27 (CI 95%: 0.05 -0.82)	NA

Gout	1 (0)	0.16 (CI 95%: 0.03 -0.93)	2 (0)	0.39 (CI 95%: 0.11 -1.41)	3 (0)	0.27 (CI 95%: 0.05 -0.82)	NA
Osteopenia	1 (0)	0.16 (CI 95%: 0.03 -0.93)	2 (0)	0.39 (CI 95%: 0.11 -1.41)	3 (0)	0.27 (CI 95%: 0.05 -0.82)	NA
CHD	1 (0)	0.16(CI 95%:0.03 -0.93)	2 (0)	0.39(CI 95%:0.11 -1.41)	3 (0)	0.27(CI 95%:0.05 -0.82)	NA
Bowel syndrome	1 (0)	0.16 (CI 95%: 0.03 -0.93)	1 (0)	0.19 (CI 95%: 0.03 -1.1)	2 (0)	0.18 (CI 95%: 0 -0.69)	NA
S/P spontaneous pneumothorax	1 (0)	0.16 (CI 95%: 0.03 -0.93)	1 (0)	0.19 (CI 95%: 0.03 -1.1)	2 (0)	0.18 (CI 95%: 0 -0.69)	NA
Obesity	1 (0)	0.16 (CI 95%: 0.03 -0.93)	1 (0)	0.19 (CI 95%: 0.03 -1.1)	2 (0)	0.18 (CI 95%: 0 -0.69)	NA
Vasculitis	1 (0)	0.16 (CI 95%: 0.03 -0.93)	1 (0)	0.19 (CI 95%: 0.03 -1.1)	2 (0)	0.18 (CI 95%: 0 -0.69)	NA
Tinnitus	1 (0)	0.16 (CI 95%: 0.03 -0.93)	1 (0)	0.19 (CI 95%: 0.03 -1.1)	2 (0)	0.18 (CI 95%: 0 -0.69)	NA
Nephrolithiasis	1 (0)	0.16 (CI 95%: 0.03 -0.93)	1 (0)	0.19 (CI 95%: 0.03 -1.1)	2 (0)	0.18 (CI 95%: 0 -0.69)	NA
AV block	1 (0)	0.16(CI 95%:0.03 -0.93)	1 (0)	0.19(CI 95%:0.03 -1.1)	2 (0)	0.18(CI 95%:0 -0.69)	NA
Pulmonary fibrosis	1 (0)	0.16(CI 95%:0.03 -0.93)	1 (0)	0.19(CI 95%:0.03 -1.1)	2 (0)	0.18(CI 95%:0 -0.69)	NA
Schizophrenia	1 (0)	0.16 (CI 95%:0.03 -0.93)	1 (0)	0.19 (CI 95%:0.03 -1.1)	2 (0)	0.18 (CI 95%:0 -0.69)	NA

Cholelithiasis	1 (0)	0.16 (CI 95%: 0.03 -0.93)	0 (0)	0 (CI 95%: 0 - 0.74)	1 (0)	0.09 (CI 95%: -0.04 -0.56)	NA
Hiatus hernia	1 (0)	0.16 (CI 95%: 0.03 -0.93)	0 (0)	0 (CI 95%: 0 - 0.74)	1 (0)	0.09 (CI 95%: -0.04 -0.56)	NA
GERD	1 (0)	0.16 (CI 95%: 0.03 -0.93)	0 (0)	0 (CI 95%: 0 - 0.74)	1 (0)	0.09 (CI 95%: -0.04 -0.56)	NA
Stomach polyp	1 (0)	0.16 (CI 95%: 0.03 -0.93)	0 (0)	0 (CI 95%: 0 - 0.74)	1 (0)	0.09 (CI 95%: -0.04 -0.56)	NA
Melanoma	1 (0)	0.16 (CI 95%: 0.03 -0.93)	0 (0)	0 (CI 95%: 0 - 0.74)	1 (0)	0.09 (CI 95%: -0.04 -0.56)	NA
Macular degeneration	1 (0)	0.16 (CI 95%: 0.03 -0.93)	0 (0)	0 (CI 95%: 0 - 0.74)	1 (0)	0.09 (CI 95%: -0.04 -0.56)	NA
Lupus	1 (0)	0.16 (CI 95%: 0.03 -0.93)	0 (0)	0 (CI 95%: 0 - 0.74)	1 (0)	0.09 (CI 95%: -0.04 -0.56)	NA
Thalassemia minor	1 (0)	0.16 (CI 95%: 0.03 -0.93)	0 (0)	0 (CI 95%: 0 - 0.74)	1 (0)	0.09 (CI 95%: -0.04 -0.56)	NA
Gall bladder stones	1 (0)	0.16 (CI 95%: 0.03 -0.93)	0 (0)	0 (CI 95%: 0 - 0.74)	1 (0)	0.09 (CI 95%: -0.04 -0.56)	NA
Duodenal ulcer	1 (0)	0.16 (CI 95%: 0.03 -0.93)	0 (0)	0 (CI 95%: 0 - 0.74)	1 (0)	0.09 (CI 95%: -0.04 -0.56)	NA
Biliary Atresia	1 (0)	0.16 (CI 95%: 0.03 -0.93)	0 (0)	0 (CI 95%: 0 - 0.74)	1 (0)	0.09 (CI 95%: -0.04 -0.56)	NA
Goiter	1 (0)	0.16 (CI 95%: 0.03 -0.93)	0 (0)	0 (CI 95%: 0 - 0.74)	1 (0)	0.09 (CI 95%: -0.04 -0.56)	NA
Kyphosis	1 (0)	0.16 (CI 95%: 0.03 -0.93)	0 (0)	0 (CI 95%: 0 - 0.74)	1 (0)	0.09 (CI 95%: -0.04 -0.56)	NA
Benign tumor	1 (0)	0.16 (CI 95%: 0.03 -0.93)	0 (0)	0 (CI 95%: 0 - 0.74)	1 (0)	0.09 (CI 95%: -0.04 -0.56)	NA

ITP	1 (0)	0.16 (CI 95%: 0.03 -0.93)	0 (0)	0 (CI 95%: 0 - 0.74)	1 (0)	0.09 (CI 95%: -0.04 -0.56)	NA
Urticaria	1 (0)	0.16 (CI 95%: 0.03 -0.93)	0 (0)	0 (CI 95%: 0 - 0.74)	1 (0)	0.09 (CI 95%: -0.04 -0.56)	NA
Polycystic ovaries removal	1 (0)	0.16 (CI 95%: 0.03 -0.93)	0 (0)	0 (CI 95%: 0 - 0.74)	1 (0)	0.09 (CI 95%: -0.04 -0.56)	NA
S/P colon and thyroid cancer	1 (0)	0.16 (CI 95%: 0.03 -0.93)	0 (0)	0 (CI 95%: 0 - 0.74)	1 (0)	0.09 (CI 95%: -0.04 -0.56)	NA
Diverticulitis	1 (0)	0.16 (CI 95%: 0.03 -0.93)	0 (0)	0 (CI 95%: 0 - 0.74)	1 (0)	0.09 (CI 95%: -0.04 -0.56)	NA
Bladder problems	1 (0)	0.16 (CI 95%: 0.03 -0.93)	0 (0)	0 (CI 95%: 0 - 0.74)	1 (0)	0.09 (CI 95%: -0.04 -0.56)	NA
Hemodialysis	1 (0)	0.16 (CI 95%: 0.03 -0.93)	0 (0)	0 (CI 95%: 0 - 0.74)	1 (0)	0.09 (CI 95%: -0.04 -0.56)	NA
Dwarfism	1 (0)	0.16 (CI 95%: 0.03 -0.93)	0 (0)	0 (CI 95%: 0 - 0.74)	1 (0)	0.09 (CI 95%: -0.04 -0.56)	NA
Femoral-facial syndrome	1 (0)	0.16 (CI 95%: 0.03 -0.93)	0 (0)	0 (CI 95%: 0 - 0.74)	1 (0)	0.09 (CI 95%: -0.04 -0.56)	NA
Chronic sinusitis	1 (0)	0.16 (CI 95%: 0.03 -0.93)	0 (0)	0 (CI 95%: 0 - 0.74)	1 (0)	0.09 (CI 95%: -0.04 -0.56)	NA
Ovarian cyst	1 (0)	0.16 (CI 95%: 0.03 -0.93)	0 (0)	0 (CI 95%: 0 - 0.74)	1 (0)	0.09 (CI 95%: -0.04 -0.56)	NA
Post nasal drip	1 (0)	0.16 (CI 95%: 0.03 -0.93)	0 (0)	0 (CI 95%: 0 - 0.74)	1 (0)	0.09 (CI 95%: -0.04 -0.56)	NA
Arachnoiditis	1 (0)	0.16 (CI 95%: 0.03 -0.93)	0 (0)	0 (CI 95%: 0 - 0.74)	1 (0)	0.09 (CI 95%: -0.04 -0.56)	NA
Polio	1 (0)	0.16 (CI 95%: 0.03 -0.93)	0 (0)	0 (CI 95%: 0 - 0.74)	1 (0)	0.09 (CI 95%: -0.04 -0.56)	NA

Aphasia	1 (0)	0.16 (CI 95%: 0.03 -0.93)	0 (0)	0 (CI 95%: 0 - 0.74)	1 (0)	0.09 (CI 95%: -0.04 -0.56)	NA
Polycythemia	1 (0)	0.16 (CI 95%: 0.03 -0.93)	0 (0)	0 (CI 95%: 0 - 0.74)	1 (0)	0.09 (CI 95%: -0.04 -0.56)	NA
APLA	1 (0)	0.16 (CI 95%: 0.03 -0.93)	0 (0)	0 (CI 95%: 0 - 0.74)	1 (0)	0.09 (CI 95%: -0.04 -0.56)	NA
White robe syndrome	1 (0)	0.16 (CI 95%: 0.03 -0.93)	0 (0)	0 (CI 95%: 0 - 0.74)	1 (0)	0.09 (CI 95%: -0.04 -0.56)	NA
Keratoconjunctivitis	1 (0)	0.16 (CI 95%: 0.03 -0.93)	0 (0)	0 (CI 95%: 0 - 0.74)	1 (0)	0.09 (CI 95%: -0.04 -0.56)	NA
Kidney stones	1 (0)	0.16 (CI 95%: 0.03 -0.93)	0 (0)	0 (CI 95%: 0 - 0.74)	1 (0)	0.09 (CI 95%: -0.04 -0.56)	NA
Helicobacter Pylori	1 (0)	0.16 (CI 95%: 0.03 -0.93)	0 (0)	0 (CI 95%: 0 - 0.74)	1 (0)	0.09 (CI 95%: -0.04 -0.56)	NA
BRCA2 carrier	1 (0)	0.16 (CI 95%: 0.03 -0.93)	0 (0)	0 (CI 95%: 0 - 0.74)	1 (0)	0.09 (CI 95%: -0.04 -0.56)	NA
Narcolepsy	1 (0)	0.16 (CI 95%: 0.03 -0.93)	0 (0)	0 (CI 95%: 0 - 0.74)	1 (0)	0.09 (CI 95%: -0.04 -0.56)	NA
Past sarcoma	1 (0)	0.16 (CI 95%: 0.03 -0.93)	0 (0)	0 (CI 95%: 0 - 0.74)	1 (0)	0.09 (CI 95%: -0.04 -0.56)	NA
S/P non-Hodgkin's lymphoma	1 (0)	0.16 (CI 95%: 0.03 -0.93)	0 (0)	0 (CI 95%: 0 - 0.74)	1 (0)	0.09 (CI 95%: -0.04 -0.56)	NA
Valve replacement	1 (0)	0.16(CI 95%:0.03 - 0.93)	0 (0)	0(CI 95%:0 - 0.74)	1 (0)	0.09(CI 95%:- 0.04 -0.56)	NA
Bradycardia	1 (0)	0.16(CI 95%:0.03 - 0.93)	0 (0)	0(CI 95%:0 - 0.74)	1 (0)	0.09(CI 95%:- 0.04 -0.56)	NA

Blood circulation disorder	1 (0)	0.16(CI 95%:0.03 - 0.93)	0 (0)	0(CI 95%:0 - 0.74)	1 (0)	0.09(CI 95%:-0.04 -0.56)	NA
S/P Myocarditis	1 (0)	0.16(CI 95%:0.03 - 0.93)	0 (0)	0(CI 95%:0 - 0.74)	1 (0)	0.09(CI 95%:-0.04 -0.56)	NA
POTS	1 (0)	0.16(CI 95%:0.03 - 0.93)	0 (0)	0(CI 95%:0 - 0.74)	1 (0)	0.09(CI 95%:-0.04 -0.56)	NA
RT Carotid full block	1 (0)	0.16(CI 95%:0.03 - 0.93)	0 (0)	0(CI 95%:0 - 0.74)	1 (0)	0.09(CI 95%:-0.04 -0.56)	NA
Radiation treatment AE	1 (0)	0.16(CI 95%:0.03 - 0.93)	0 (0)	0(CI 95%:0 - 0.74)	1 (0)	0.09(CI 95%:-0.04 -0.56)	NA
Emphysema	1 (0)	0.16(CI 95%:0.03 - 0.93)	0 (0)	0(CI 95%:0 - 0.74)	1 (0)	0.09(CI 95%:-0.04 -0.56)	NA
Lung GVHD	1 (0)	0.16(CI 95%:0.03 - 0.93)	0 (0)	0(CI 95%:0 - 0.74)	1 (0)	0.09(CI 95%:-0.04 -0.56)	NA
Hypomania	1 (0)	0.16 (CI 95%:0.03 - 0.93)	0 (0)	0 (CI 95%:0 - 0.74)	1 (0)	0.09 (CI 95%:-0.04 - 0.56)	NA
Addiction	1 (0)	0.16 (CI 95%:0.03 - 0.93)	0 (0)	0 (CI 95%:0 - 0.74)	1 (0)	0.09 (CI 95%:-0.04 - 0.56)	NA
BPH	0 (0)	0 (CI 95%: 0 - 0.63)	37 (7)	7.21 (CI 95%: 5.28 -9.78)	37 (3)	3.3 (CI 95%: 2.39 -4.53)	NA

Coronary stent	0 (0)	0(CI 95%:0 - 0.63)	4 (1)	0.78(CI 95%:0.3 -1.99)	4 (0)	0.36(CI 95%:0.1 - 0.95)	NA
Dry mouth	0 (0)	0 (CI 95%: 0 - 0.63)	2 (0)	0.39 (CI 95%: 0.11 -1.41)	2 (0)	0.18 (CI 95%: 0 -0.69)	NA
Cirrhosis	0 (0)	0 (CI 95%: 0 - 0.63)	2 (0)	0.39 (CI 95%: 0.11 -1.41)	2 (0)	0.18 (CI 95%: 0 -0.69)	NA
Hernia	0 (0)	0 (CI 95%: 0 - 0.63)	2 (0)	0.39 (CI 95%: 0.11 -1.41)	2 (0)	0.18 (CI 95%: 0 -0.69)	NA
Irritable bladder	0 (0)	0 (CI 95%: 0 - 0.63)	2 (0)	0.39 (CI 95%: 0.11 -1.41)	2 (0)	0.18 (CI 95%: 0 -0.69)	NA
Hyperparathyroidism	0 (0)	0 (CI 95%: 0 - 0.63)	2 (0)	0.39 (CI 95%: 0.11 -1.41)	2 (0)	0.18 (CI 95%: 0 -0.69)	NA
Bypass surgery	0 (0)	0(CI 95%:0 - 0.63)	3 (1)	0.58(CI 95%:0.2 -1.71)	3 (0)	0.27(CI 95%:0.05 - 0.82)	NA
Cardiomyopathy	0 (0)	0(CI 95%:0 - 0.63)	2 (0)	0.39(CI 95%:0.11 - 1.41)	2 (0)	0.18(CI 95%:0 -0.69)	NA
OCD	0 (0)	0 (CI 95%:0 - 0.63)	3 (1)	0.58 (CI 95%:0.2 -1.71)	3 (0)	0.27 (CI 95%:0.05 - 0.82)	NA
Parathyroidectomy	0 (0)	0 (CI 95%: 0 - 0.63)	1 (0)	0.19 (CI 95%: 0.03 -1.1)	1 (0)	0.09 (CI 95%: -0.04 -0.56)	NA
S/P Knee surgeries	0 (0)	0 (CI 95%: 0 - 0.63)	1 (0)	0.19 (CI 95%: 0.03 -1.1)	1 (0)	0.09 (CI 95%: -0.04 -0.56)	NA
S/P B cell lymphoma	0 (0)	0 (CI 95%: 0 - 0.63)	1 (0)	0.19 (CI 95%: 0.03 -1.1)	1 (0)	0.09 (CI 95%: -0.04 -0.56)	NA

S/P complicated bariatric surgery	0 (0)	0 (CI 95%: 0 - 0.63)	1 (0)	0.19 (CI 95%: 0.03 -1.1)	1 (0)	0.09 (CI 95%: -0.04 -0.56)	NA
Churg-Strauss syndrome	0 (0)	0 (CI 95%: 0 - 0.63)	1 (0)	0.19 (CI 95%: 0.03 -1.1)	1 (0)	0.09 (CI 95%: -0.04 -0.56)	NA
Addison's disease	0 (0)	0 (CI 95%: 0 - 0.63)	1 (0)	0.19 (CI 95%: 0.03 -1.1)	1 (0)	0.09 (CI 95%: -0.04 -0.56)	NA
Alzheimer's disease	0 (0)	0 (CI 95%: 0 - 0.63)	1 (0)	0.19 (CI 95%: 0.03 -1.1)	1 (0)	0.09 (CI 95%: -0.04 -0.56)	NA
Lobectomy	0 (0)	0 (CI 95%: 0 - 0.63)	1 (0)	0.19 (CI 95%: 0.03 -1.1)	1 (0)	0.09 (CI 95%: -0.04 -0.56)	NA
Lung cancer	0 (0)	0 (CI 95%: 0 - 0.63)	1 (0)	0.19 (CI 95%: 0.03 -1.1)	1 (0)	0.09 (CI 95%: -0.04 -0.56)	NA
Chronic prostatitis	0 (0)	0 (CI 95%: 0 - 0.63)	1 (0)	0.19 (CI 95%: 0.03 -1.1)	1 (0)	0.09 (CI 95%: -0.04 -0.56)	NA
Uveitis	0 (0)	0 (CI 95%: 0 - 0.63)	1 (0)	0.19 (CI 95%: 0.03 -1.1)	1 (0)	0.09 (CI 95%: -0.04 -0.56)	NA
Drop foot	0 (0)	0 (CI 95%: 0 - 0.63)	1 (0)	0.19 (CI 95%: 0.03 -1.1)	1 (0)	0.09 (CI 95%: -0.04 -0.56)	NA
S/P Lymphoma	0 (0)	0 (CI 95%: 0 - 0.63)	1 (0)	0.19 (CI 95%: 0.03 -1.1)	1 (0)	0.09 (CI 95%: -0.04 -0.56)	NA
Meniere's disease	0 (0)	0 (CI 95%: 0 - 0.63)	1 (0)	0.19 (CI 95%: 0.03 -1.1)	1 (0)	0.09 (CI 95%: -0.04 -0.56)	NA
Astigmatism	0 (0)	0 (CI 95%: 0 - 0.63)	1 (0)	0.19 (CI 95%: 0.03 -1.1)	1 (0)	0.09 (CI 95%: -0.04 -0.56)	NA
Infertility	0 (0)	0 (CI 95%: 0 - 0.63)	1 (0)	0.19 (CI 95%: 0.03 -1.1)	1 (0)	0.09 (CI 95%: -0.04 -0.56)	NA
Splenomegaly	0 (0)	0 (CI 95%: 0 - 0.63)	1 (0)	0.19 (CI 95%: 0.03 -1.1)	1 (0)	0.09 (CI 95%: -0.04 -0.56)	NA

Hypogonadism	0 (0)	0 (CI 95%: 0 - 0.63)	1 (0)	0.19 (CI 95%: 0.03 -1.1)	1 (0)	0.09 (CI 95%: -0.04 -0.56)	NA
Pre-dialysis	0 (0)	0 (CI 95%: 0 - 0.63)	1 (0)	0.19 (CI 95%: 0.03 -1.1)	1 (0)	0.09 (CI 95%: -0.04 -0.56)	NA
Hydronephrotic	0 (0)	0 (CI 95%: 0 - 0.63)	1 (0)	0.19 (CI 95%: 0.03 -1.1)	1 (0)	0.09 (CI 95%: -0.04 -0.56)	NA
Brain metastases	0 (0)	0 (CI 95%: 0 - 0.63)	1 (0)	0.19 (CI 95%: 0.03 -1.1)	1 (0)	0.09 (CI 95%: -0.04 -0.56)	NA
Gilbert's Syndrome	0 (0)	0 (CI 95%: 0 - 0.63)	1 (0)	0.19 (CI 95%: 0.03 -1.1)	1 (0)	0.09 (CI 95%: -0.04 -0.56)	NA
Spinal fracture	0 (0)	0 (CI 95%: 0 - 0.63)	1 (0)	0.19 (CI 95%: 0.03 -1.1)	1 (0)	0.09 (CI 95%: -0.04 -0.56)	NA
VF	0 (0)	0(CI 95%:0 - 0.63)	1 (0)	0.19(CI 95%:0.03 -1.1)	1 (0)	0.09(CI 95%:- 0.04 -0.56)	NA
Aortic dilatation	0 (0)	0(CI 95%:0 - 0.63)	1 (0)	0.19(CI 95%:0.03 -1.1)	1 (0)	0.09(CI 95%:- 0.04 -0.56)	NA
Aortic regurgitation	0 (0)	0(CI 95%:0 - 0.63)	1 (0)	0.19(CI 95%:0.03 -1.1)	1 (0)	0.09(CI 95%:- 0.04 -0.56)	NA
Pulmonary edema	0 (0)	0(CI 95%:0 - 0.63)	1 (0)	0.19(CI 95%:0.03 -1.1)	1 (0)	0.09(CI 95%:- 0.04 -0.56)	NA
S/P Pulmonary effusion	0 (0)	0(CI 95%:0 - 0.63)	1 (0)	0.19(CI 95%:0.03 -1.1)	1 (0)	0.09(CI 95%:- 0.04 -0.56)	NA
Myasthenia Gravis	0 (0)	0(CI 95%:0 - 0.63)	1 (0)	0.19(CI 95%:0.03 -1.1)	1 (0)	0.09(CI 95%:- 0.04 -0.56)	NA
Lung injury	0 (0)	0(CI 95%:0 - 0.63)	1 (0)	0.19(CI 95%:0.03 -1.1)	1 (0)	0.09(CI 95%:- 0.04 -0.56)	NA
Smoking lung damage	0 (0)	0(CI 95%:0 - 0.63)	1 (0)	0.19(CI 95%:0.03 -1.1)	1 (0)	0.09(CI 95%:- 0.04 -0.56)	NA

Tonsil's enlargement	0 (0)	0(CI 95%:0 - 0.63)	1 (0)	0.19(CI 95%:0.03 -1.1)	1 (0)	0.09(CI 95%:- 0.04 -0.56)	NA
Suicidal ideation	0 (0)	0 (CI 95%:0 - 0.63)	1 (0)	0.19 (CI 95%:0.03 -1.1)	1 (0)	0.09 (CI 95%:-0.04 - 0.56)	NA

ADHD, attention deficit hyperactivity disorder; AE, adverse event; AF, atrial fibrillation; AV block, atrioventricular block; BPH, benign prostatic hyperplasia; CHD coronary heart disease; CHF chronic heart failure; COPD chronic obstructive pulmonary disease; CRF chronic renal failure; CVA, cerebrovascular accident; DVT, deep vein thrombosis; FMF, familiar Mediterranean fever; GERD, gastro-esophageal reflux disease; HTN, hypertension; HCV, hepatitis C virus; IHD, ischemic heart disease; ITP, immune thrombocytopenic purpura; OCD, obsessive compulsive disorder; OSA, obstructive sleep apnea; PE, pulmonary emboli; PTCA, percutaneous transluminal coronary angioplasty; POTS, postural tachycardia syndrome; PVD, peripheral vascular disease; S/P status post; SVT, supraventricular tachycardia; NA, not applicable; Numbers do not add up to 100% due to concomitant diagnoses.

Supplement Table 6: Patients' reported treatment-related specific AEs by sex

	Analyzed population N=1249			Statistic
**Adverse events by SOC**	**Proportion in Women (CI 95%) N=666**	**Proportion in Men (CI 95%) N=582**	**Total (CI 95%) N=1248**	**Wilson's X^2 test (p-value)**
**Any (at least 1 AE)**	10.02 (CI 95%: 8.47 - 11.81)	9.76 (CI 95%: 7.73 - 12.25)	10.31 (CI 95%: 8.09 - 13.05)	0.05(0.82)
**Nervous System Disorders**				
Dizziness (Excl Vertigo)	4.49 (CI 95%: 3.47 - 5.78)	3.3 (CI 95%: 2.19 - 4.95)	3.95 (CI 95%: 2.65 - 5.86)	0.21(0.65)
Headache	3.12 (CI 95%: 2.29 - 4.24)	2.25 (CI 95%: 1.37 - 3.68)	2.75 (CI 95%: 1.7 - 4.42)	0.15(0.7)
Weakness Of Limbs	0.88 (CI 95%: 0.49 - 1.57)	0.9 (CI 95%: 0.41 - 1.95)	0.86 (CI 95%: 0.37 - 2)	0(1)
Memory Impairment	0.32 (CI 95%: 0.12 - 0.82)	0.45 (CI 95%: 0.15 - 1.32)	0.17 (CI 95%: 0.03 - 0.97)	0.14(0.71)
Balance Impaired Nos	0.24 (CI 95%: 0.08 - 0.7)	0.3 (CI 95%: 0.08 - 1.09)	0.17 (CI 95%: 0.03 - 0.97)	0(1)
Blurred Vision	0.16 (CI 95%: 0.04 - 0.58)	0.15 (CI 95%: 0.03 - 0.85)	0.17 (CI 95%: 0.03 - 0.97)	0(1)
Burning Sensation In Face	0.08 (CI 95%: 0.01 - 0.45)	0.15 (CI 95%: 0.03 - 0.85)	0 (CI 95%: 0 - 0.66)	0(1)
Hyperalgesia	0.24 (CI 95%: 0.08 - 0.7)	0.15 (CI 95%: 0.03 - 0.85)	0.34 (CI 95%: 0.09 - 1.24)	0.01(0.91)
Burning Sensation Of Lower Limb	0.08 (CI 95%: 0.01 - 0.45)	0.15 (CI 95%: 0.03 - 0.85)	0 (CI 95%: 0 - 0.66)	0(1)
Tremor	0.08 (CI 95%: 0.01 - 0.45)	0.15 (CI 95%: 0.03 - 0.85)	0 (CI 95%: 0 - 0.66)	0(1)

	Analyzed population N=1249			Statistic
**Adverse events by SOC**	**Proportion in Women (CI 95%) N=666**	**Proportion in Men (CI 95%) N=582**	**Total (CI 95%) N=1248**	**Wilson's X^2 test (p-value)**
Bad Taste	0.16 (CI 95%: 0.04 - 0.58)	0 (CI 95%: 0 - 0.57)	0.34 (CI 95%: 0.09 - 1.24)	0.65(0.42)
Other Speech Disturbance	0.08 (CI 95%: 0.01 - 0.45)	0 (CI 95%: 0 - 0.57)	0.17 (CI 95%: 0.03 - 0.97)	0(0.95)
Ringing in Ears	0.08 (CI 95%: 0.01 - 0.45)	0 (CI 95%: 0 - 0.57)	0.17 (CI 95%: 0.03 - 0.97)	0(0.95)
**Gastrointestinal Disorders**				
Sore Throat	2.24 (CI 95%: 1.56 - 3.22)	2.7 (CI 95%: 1.72 - 4.23)	1.2 (CI 95%: 0.58 - 2.46)	2.84(0.09)
Nausea	1.12 (CI 95%: 0.67 - 1.87)	1.5 (CI 95%: 0.82 - 2.74)	0.34 (CI 95%: 0.09 - 1.24)	3.24(0.07)
Dry Mouth	1.04 (CI 95%: 0.61 - 1.77)	0.75 (CI 95%: 0.32 - 1.75)	1.03 (CI 95%: 0.47 - 2.23)	0.05(0.82)
Vomiting	0.4 (CI 95%: 0.17 - 0.93)	0.45 (CI 95%: 0.15 - 1.32)	0.17 (CI 95%: 0.03 - 0.97)	0.14(0.71)
Heartburn	0.4 (CI 95%: 0.17 - 0.93)	0.45 (CI 95%: 0.15 - 1.32)	0.34 (CI 95%: 0.09 - 1.24)	0(1)
Abdominal Pain Nos	0.08 (CI 95%: 0.01 - 0.45)	0.15 (CI 95%: 0.03 - 0.85)	0 (CI 95%: 0 - 0.66)	0(1)
**General Disorders and Administration Site Conditions**				
Sleepiness	2.4 (CI 95%: 1.69 - 3.41)	2.85 (CI 95%: 1.83 - 4.41)	1.37 (CI 95%: 0.7 - 2.69)	2.55(0.11)
Diplopia	0.08 (CI 95%: 0.01 - 0.45)	0 (CI 95%: 0 - 0.57)	0.17 (CI 95%: 0.03 - 0.97)	0(0.95)

	Analyzed population N=1249			Statistic
**Adverse events by SOC**	**Proportion in Women (CI 95%) N=666**	**Proportion in Men (CI 95%) N=582**	**Total (CI 95%) N=1248**	**Wilson's X^2 test (p-value)**
Thirst	0.08 (CI 95%: 0.01 - 0.45)	0 (CI 95%: 0 - 0.57)	0.17 (CI 95%: 0.03 - 0.97)	0(0.95)
**Respiratory, Thoracic and Mediastinal Disorders**				
Cough	2.72 (CI 95%: 1.96 - 3.78)	2.7 (CI 95%: 1.72 - 4.23)	2.41 (CI 95%: 1.44 - 4)	0.02(0.88)
Difficulty Breathing	0.32 (CI 95%: 0.12 - 0.82)	0.45 (CI 95%: 0.15 - 1.32)	0.17 (CI 95%: 0.03 - 0.97)	0.14(0.71)
Dyspnea	0.32 (CI 95%: 0.12 - 0.82)	0.45 (CI 95%: 0.15 - 1.32)	0.17 (CI 95%: 0.03 - 0.97)	0.14(0.71)
**Psychiatric Disorders**				
Abnormal Sleep-Related Events	0.64 (CI 95%: 0.33 - 1.26)	0.9 (CI 95%: 0.41 - 1.95)	0.17 (CI 95%: 0.03 - 0.97)	1.8(0.18)
Poor Quality Sleep	0.64 (CI 95%: 0.33 - 1.26)	0.9 (CI 95%: 0.41 - 1.95)	0.17 (CI 95%: 0.03 - 0.97)	1.8(0.18)
Night Sweats	0.64 (CI 95%: 0.33 - 1.26)	0.9 (CI 95%: 0.41 - 1.95)	0.17 (CI 95%: 0.03 - 0.97)	1.8(0.18)
Anxiety	0.72 (CI 95%: 0.38 - 1.36)	0.6 (CI 95%: 0.23 - 1.53)	0.52 (CI 95%: 0.18 - 1.5)	0(1)
Impairment of Attention	0.16 (CI 95%: 0.04 - 0.58)	0.15 (CI 95%: 0.03 - 0.85)	0.17 (CI 95%: 0.03 - 0.97)	0(1)
Bad Mood	0.08 (CI 95%: 0.01 - 0.45)	0.15 (CI 95%: 0.03 - 0.85)	0 (CI 95%: 0 - 0.66)	0(1)
Disrupted Thoughts	0.16 (CI 95%: 0.04 - 0.58)	0.15 (CI 95%: 0.03 - 0.85)	0.17 (CI 95%: 0.03 - 0.97)	0(1)

	Analyzed population<br>N=1249			Statistic
Adverse events by SOC	Proportion in Women<br>(CI 95%)<br>N=666	Proportion in Men<br>(CI 95%)<br>N=582	Total<br>(CI 95%)<br>N=1248	Wilson's X^2 test<br>(p-value)
Nervousness	0.56 (CI 95%: 0.27 - 1.15)	0.15 (CI 95%: 0.03 - 0.85)	0.86 (CI 95%: 0.37 - 2)	1.95(0.16)
Euphoria	0.08 (CI 95%: 0.01 - 0.45)	0 (CI 95%: 0 - 0.57)	0.17 (CI 95%: 0.03 - 0.97)	0(0.95)
Confusion	0.4 (CI 95%: 0.17 - 0.93)	0 (CI 95%: 0 - 0.57)	0.69 (CI 95%: 0.27 - 1.75)	2.69(0.1)
**Cardiac Disorders**				
Palpitations	0.72 (CI 95%: 0.38 - 1.36)	0.45 (CI 95%: 0.15 - 1.32)	0.86 (CI 95%: 0.37 - 2)	0.3(0.58)
**Musculoskeletal and Connective Tissue Disorders**				
Motor Dysfunction Nos	0.08 (CI 95%: 0.01 - 0.45)	0 (CI 95%: 0 - 0.57)	0.17 (CI 95%: 0.03 - 0.97)	0(0.95)
**Skin and Subcutaneous Tissue Disorders**				
Sweaty	0.08 (CI 95%: 0.01 - 0.45)	0 (CI 95%: 0 - 0.57)	0.17 (CI 95%: 0.03 - 0.97)	0(0.95)
**Vascular Disorders**				
Hypotension	0.16 (CI 95%: 0.04 - 0.58)	0 (CI 95%: 0 - 0.57)	0.34 (CI 95%: 0.09 - 1.24)	0.65(0.42)

AEs, Adverse events; CI, Confidence interval; PT, Preferred term; SOC, System organ classification; Numbers do not add up to 100% due to concomitant AEs.

Results S1: Pain reduction response comparison by sex

For the general sample, at the end of the up-titration phase, no significant differences were found between women and men in treatment effectiveness. At D_{30}, when pain reduction started to plateau, 348 patients (68%) reported a reduction in pain intensity of at least one numerical pain scale point (of them, n=202, 58% were women and n=146, 42% were men), 135 patients (26%) reported no change (of them, n=71, 53% were women and n=64, 47% were men), and 28 patients (5%) reported an increase in pain intensity (of them, n=14, 50% were women and n=14, 50% were men) ($\chi^2_{(1)}$=1.60, p=0.44). In addition, At D_{30}, there were no significant differences in the magnitude of pain intensity reduction between women and men. Overall, 132 patients (26%) reported a reduction of ≥30% (n=67, 23% for women and n=65, 29% for men; $\chi^2_{(1)}$=1.90, p=0.17) and 54 patients (11%) reported a reduction of ≥50% from D_0 (n=27, 9% for women and n=27, 12% for men; $\chi^2_{(1)}$=0.70, p=0.40). Thus, no clinical difference in the response to the treatment have been found between the sexes.

Among the 28 patients who reported an increase in pain intensity following a 30-days treatment period with the MDI, 12 (44%) patients provided data on the following secondary outcomes: sleep duration, sleep quality, McGill score and health-related quality of life score. From these patients, nine (32%) reported sleep duration improvement, six (22%) patients reported sleep quality improvement, four (14%) reported quality of life improvement, three reported McGill total score reduction (affective and sensory pain intensities) and two (7%) patients reported pain catastrophizing score improvement at D_{30} in comparison to D_0. The remaining 16 patients did not provide any additional information for the outcome measures other than weekly average pain intensity.

Among the 45 patients who reported their pain intensity at D_{30} and at D_{240} (i.e., study endpoint), by means of mixed linear regression analysis, pain intensity measured by NPS decreased by 1.58 (95% CI, −3.02 to −0.15; p<0.05), from 7.4 ± 2.0 at baseline to 5.7 ± 1.7 at D_{240} following treatment initiation (27% mean pain reduction) with no sex differences apparent. For sensitivity analysis, LOCF was performed on that measure, increasing the number of reports at D_{240} to 145 instead of 45. Mixed linear regression analysis showed that the pain intensity measured by NPS decreased by 2.60 (95% CI, −2.91 to −2.30; p<0.001) at D_{240}.

Table S7: Descriptive sample etiologies

Study arm	Placebo N=17		500 mcg THC N=17		1,000 mcg THC N=25		1,500 mcg THC N=3		Total N=25
**Pain diagnosis etiology**	**W N=3 n (%)**	**M N=14 n (%)**	**W N=3 n (%)**	**M N=14 n (%)**	**W N=6 n (%)**	**M N=19 n (%)**	**W N=2 n (%)**	**M N=1 n (%)**	**n (%)**
**Chronic pain**	3 (100)	14 (100)	3 (100)	14 (100)	6 (100)	19 (100)	2 (100)	1 (100)	25 (100)
**Neuropathic pain**	3 (100)	14 (100)	3 (100)	14 (100)	6 (100)	19 (100)	2 (100)	1 (100)	25 (100)
Lumbar radicular pain	0	3 (21)	0	3 (21)	1 (17)	4 (21)	NA	NA	5 (20)
Complex regional pain syndrome	0	4 (29)	0	4 (29)	1 (17)	3 (16)	0	1 (100)	4 (16)
Painful diabetic peripheral pain	0	3 (21)	0	3 (21)	0	3 (16)	NA	NA	3 (12)
Phantom limb pain	0	1 (7)	0	1 (7)	0	1 (5)	NA	NA	1 (4)
Nerve injury	NA	NA	NA	NA	1 (17)	0	1 (50)	0	1 (4)
Cervical radicular pain	NA	NA	NA	NA	1 (17)	0	NA	NA	1 (4)
Berger's disease	0	1 (7)	0	1 (7)	0	1 (5)	NA	NA	1 (4)
Idiopathic peripheral neuropathy	1 (33)	1 (7)	1 (33)	1 (7)	1 (17)	1 (5)	1 (50)	0	2 (8)
Failed back surgery pain	0	1 (7)	0	1 (7)	1 (17)	1 (5)	NA	NA	2 (8)
**Musculoskeletal pain**	NA	NA	NA	NA	0	1 (5)	NA	NA	1 (4)
Sacroiliitis	NA	NA	NA	NA	0	1 (5)	NA	NA	1 (4)

**Headaches**	0	1 (7)	0	1 (7)	0	1 (5)	NA	NA	1 (4)
Cluster headache	0	1 (7)	0	1 (7)	0	1 (5)	NA	NA	1 (4)
**inflammatory bowel diseases**	1 (33)	1 (7)	1 (33)	1 (7)	2 (34)	1 (5)	1 (50)	0	3 (12)
Crohn's disease	1 (33)	1 (7)	1 (33)	1 (7)	1 (17)	1 (5)	1 (50)	0	2 (8)
Ulcerative colitis	NA	NA	NA	NA	1 (17)	0	NA	NA	1 (4)

W, women; M, men; NA, not applicable; Numbers do not add up to 100% due to concomitant diagnoses.

FigureS1: Δ^9-THC and its metabolites pharmacokinetics

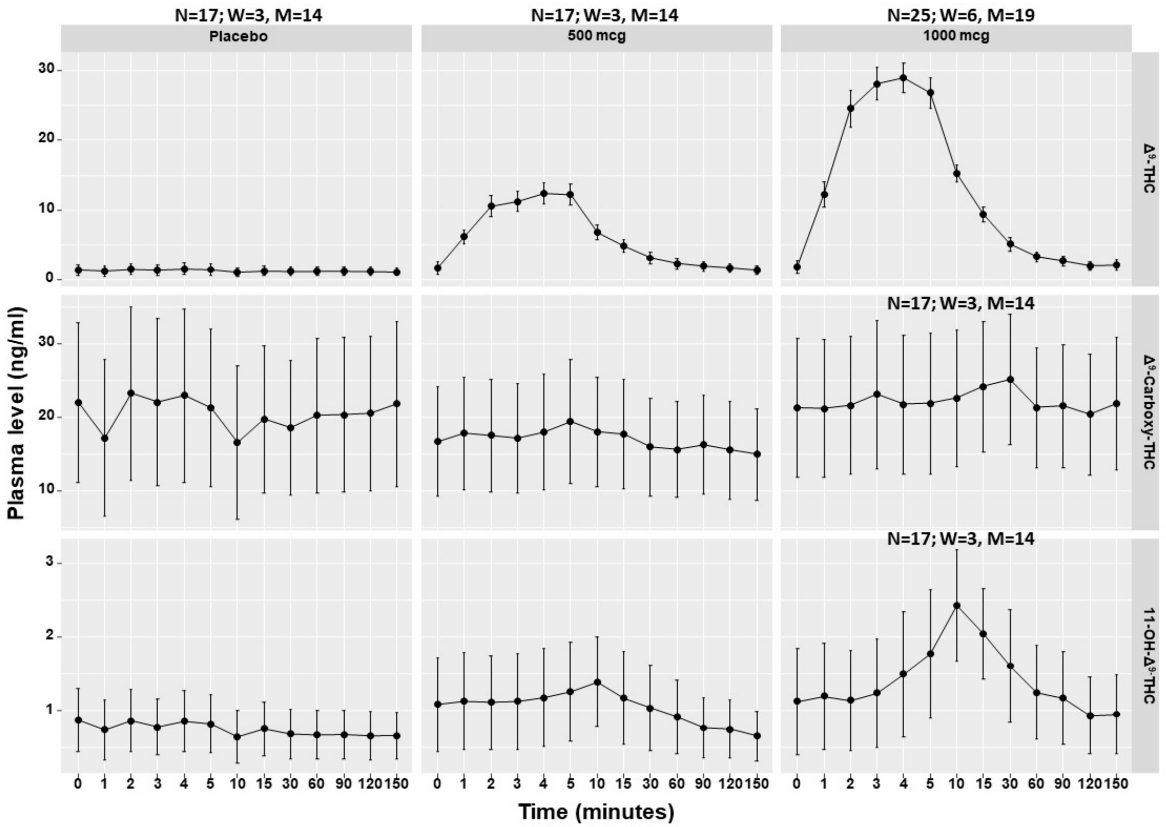

Δ^9-THC, Tetrahydrocannabinol; 11-Nor-9-carboxy-Δ9-tetrahydrocannabinol;11-OH-Δ^9-THC, 11-Nor-9-carboxy-Δ9-tetrahydrocannabinol; M, Men; W, Women, error bars represent standard deviation.

FigureS2: Individualized sex differences in Δ^{9}-THC and its metabolites pharmacokinetics

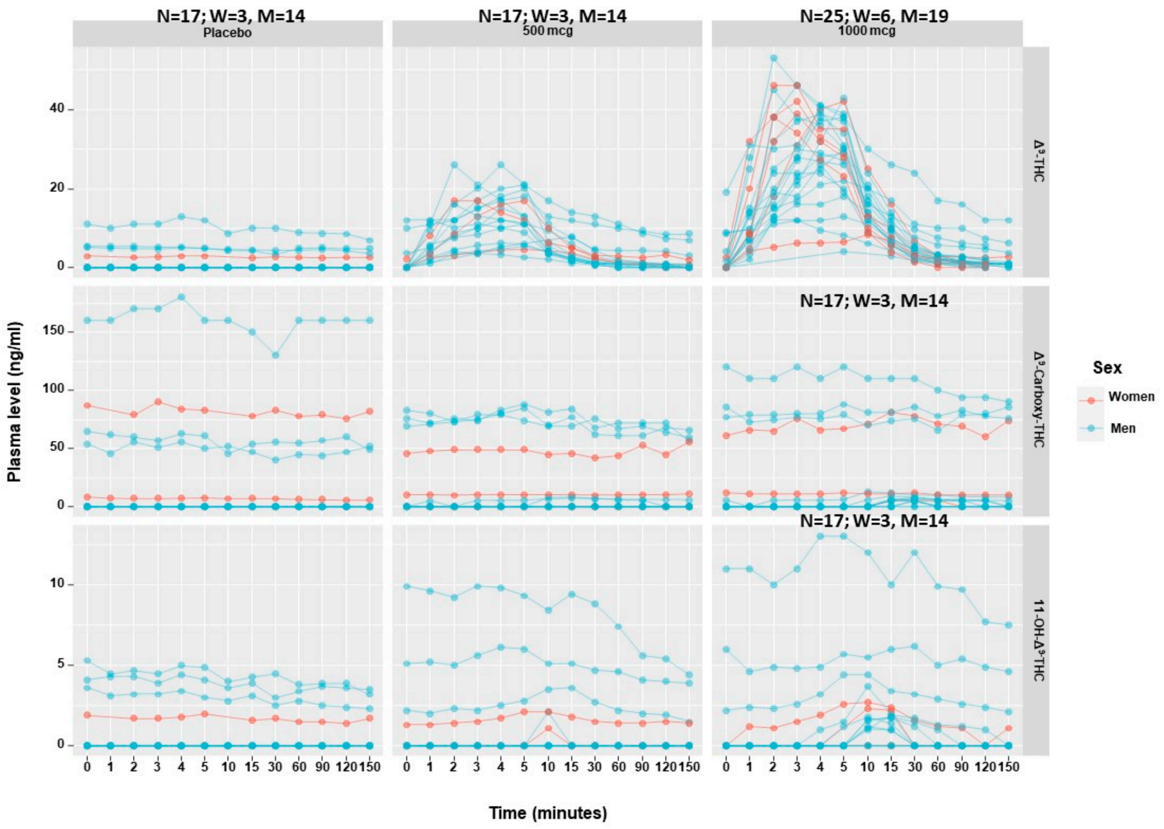

Δ^{9}-THC, Tetrahydrocannabinol; 11-Nor-9-carboxy-Δ9-tetrahydrocannabinol;11-OH-Δ^{9}-THC, 11-Nor-9-carboxy-Δ9-tetrahydrocannabinol; M, Men; W, Women. Notably, neither of the sexes exhibited outlier behavior that might have impacted the results of the comparison.

FigureS3: Sex comparison of blood pressure following a single inhalation

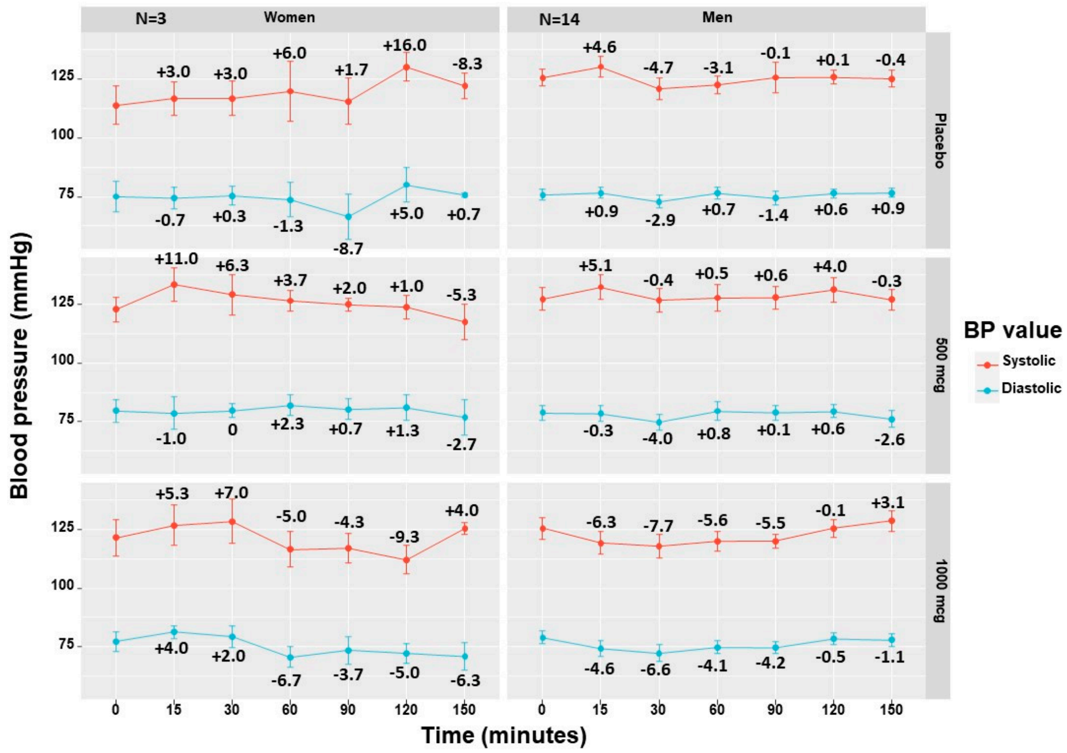

BP, Blood pressure, error bars represent standard deviation.

FigureS4: Sex comparison of heart rate following a single inhalation

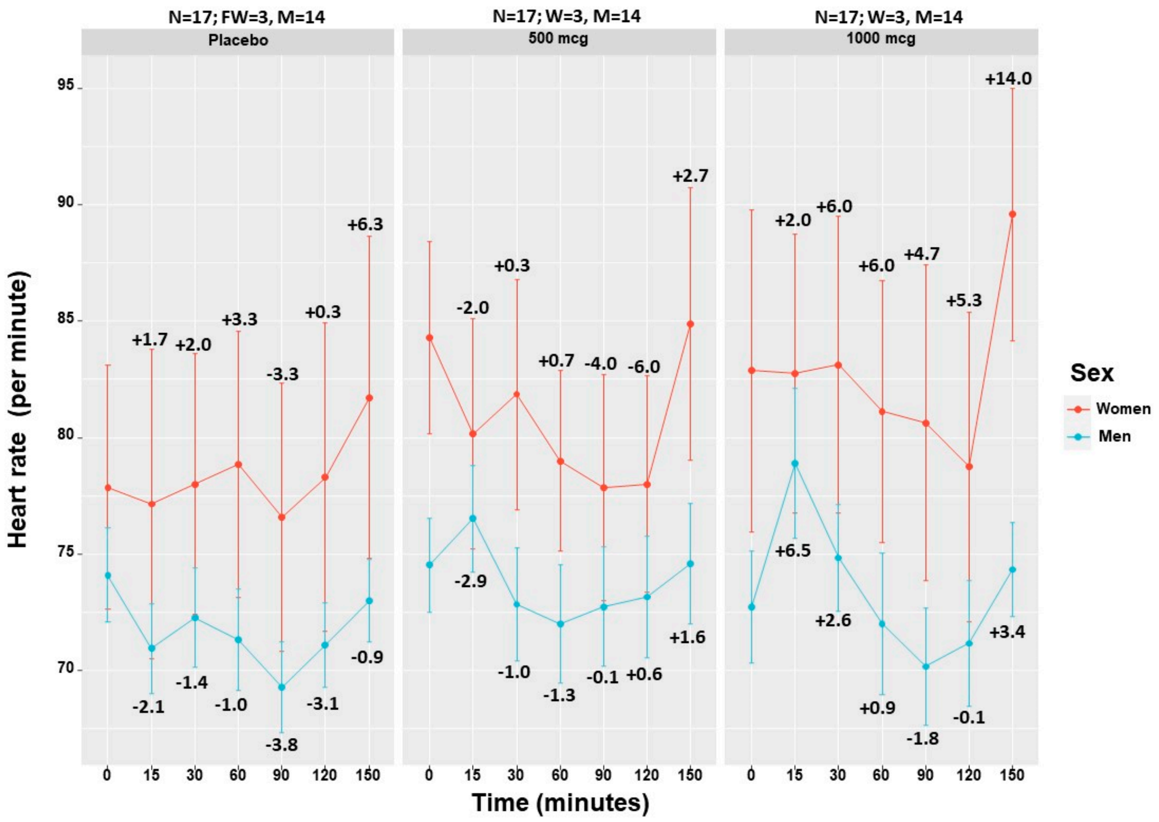

M, Men; W, Women, error bars represent standard deviation.
